# Phase 1 Trial of AMA1-C1/Alhydrogel plus CPG 7909: An Asexual Blood-Stage Vaccine for *Plasmodium falciparum* Malaria

**DOI:** 10.1371/journal.pone.0002940

**Published:** 2008-08-13

**Authors:** Gregory E. D. Mullen, Ruth D. Ellis, Kazutoyo Miura, Elissa Malkin, Caroline Nolan, Mhorag Hay, Michael P. Fay, Allan Saul, Daming Zhu, Kelly Rausch, Samuel Moretz, Hong Zhou, Carole A. Long, Louis H. Miller, John Treanor

**Affiliations:** 1 Malaria Vaccine Development Branch, National Institute of Allergy and Infectious Diseases, National Institutes of Health, Rockville, Maryland, United States of America; 2 PATH Malaria Vaccine Initiative, Bethesda, Maryland, United States of America; 3 Department of Medicine, University of Rochester, Rochester, New York, United States of America; 4 Biostatistics Research Branch, National Institute of Allergy and Infectious Diseases, National Institutes of Health, Bethesda, Maryland, United States of America; 5 Novartis Vaccines Institute for Global Health S.r.l. (NVGH), Siena, Italy; 6 Laboratory of Malaria and Vector Research, National Institute of Allergy and Infectious Diseases, National Institutes of Health, Rockville, Maryland, United States of America; New England Biolabs, United States of America

## Abstract

**Background:**

Apical Membrane Antigen 1 (AMA1), a polymorphic merozoite surface protein, is a leading blood-stage malaria vaccine candidate. This is the first reported use in humans of an investigational vaccine, AMA1-C1/Alhydrogel, with the novel adjuvant CPG 7909.

**Methods:**

A phase 1 trial was conducted at the University of Rochester with 75 malaria-naive volunteers to assess the safety and immunogenicity of the AMA1-C1/Alhydrogel+CPG 7909 malaria vaccine. Participants were sequentially enrolled and randomized within dose escalating cohorts to receive three vaccinations on days 0, 28 and 56 of either 20 µg of AMA1-C1/Alhydrogel®+564 µg CPG 7909 (n = 15), 80 µg of AMA1-C1/Alhydrogel® (n = 30), or 80 µg of AMA1-C1/Alhydrogel+564 µg CPG 7909 (n = 30).

**Results:**

Local and systemic adverse events were significantly more likely to be of higher severity with the addition of CPG 7909. Anti-AMA1 immunoglobulin G (IgG) were detected by enzyme-linked immunosorbent assay (ELISA), and the immune sera of volunteers that received 20 µg or 80 µg of AMA1-C1/Alhydrogel+CPG 7909 had up to 14 fold significant increases in anti-AMA1 antibody concentration compared to 80 µg of AMA1-C1/Alhydrogel alone. The addition of CPG 7909 to the AMA1-C1/Alhydrogel vaccine in humans also elicited AMA1 specific immune IgG that significantly and dramatically increased the in vitro growth inhibition of homologous parasites to levels as high as 96% inhibition.

**Conclusion/Significance:**

The safety profile of the AMA1-C1/Alhydrogel+CPG 7909 malaria vaccine is acceptable, given the significant increase in immunogenicity observed. Further clinical development is ongoing.

**Trial Registration:**

ClinicalTrials.gov NCT00344539

## Introduction

As reported by the World Health Organization (WHO) in 2005, the worldwide incidence of malaria is approximately 350–500 million clinical episodes annually, with more than 1 million deaths per year attributed to malaria alone and many additional deaths caused through synergy with other diseases [1]. Most of this mortality occurs among children under 5 years of age in sub-Saharan Africa. Of the 4 species of malaria that infect humans, *P. falciparum* is responsible for the majority of these deaths. Mounting drug resistance of the malaria parasite, as well as widespread resistance of mosquitoes to insecticides, make these control strategies increasingly problematic. A vaccine that would reduce both mortality and morbidity secondary to *P. falciparum* infection would be a valuable resource in the fight against this disease.

The asexual blood-stage merozoite surface antigen Apical Membrane Antigen 1 (AMA1) has been shown in several preclinical studies to be a promising malaria vaccine candidate [2]–[6]. Alignments of published *P. falciparum* AMA1 gene sequences from different isolates reveal that the AMA1 gene occurs as distinct allelic variants that differ by a number of point mutations, resulting in at least 64 known polymorphisms in the amino acid sequence [7]–[13]. Immunization with only one form of AMA1 may not protect against parasites expressing different AMA1 alleles. The combination of two or more divergent AMA1 alleles within a single vaccine formulation may provide broader protection against parasites with varying AMA1 sequences in the field, if some degree of cross-strain protection is induced [14], [15]. For this reason, the AMA1-C1 vaccine contains equal mixtures of the recombinant AMA1 proteins derived from the FVO and 3D7 strains of *P. falciparum*.

Four previous studies in the US and Mali have evaluated AMA1-C1 formulated on Alhydrogel® without CPG 7909 [16]–[19]. Some volunteers in the US Phase 1 clinical trial achieved AMA1 antibody levels that partially blocked *P. falciparum* invasion of erythrocytes; however, the majority of volunteers displayed low antibody levels with little or no *in vitro* inhibition of parasite invasion [16]. Antibody responses in semi-immune Malian adults were superior to those seen in the US, and responses were seen after the first dose [17]. We believed that higher antibody responses would be more likely to lead to protection. AMA1 has also been tested in humans in combination with other adjuvants such as AS02A, AS01B and Montanide ISA 720 [20], [21].

The novel adjuvant CPG 7909 (VaxImmune™), a CpG oligodeoxynucleotide (ODN), has been administered to humans in combination with hepatitis B vaccine, inactivated influenza vaccine, and anthrax vaccine [22]–[25]. Results from these trials indicated that, although adverse events were more frequent in some studies, [22]–[24] the addition of CPG 7909 was acceptable, and in the case of the alum-adjuvanted hepatitis B and anthrax vaccines, induced significantly higher and earlier antibody responses than the vaccine alone. Preclinical and formulation studies with CPG 7909 added to AMA1-C1/Alhydrogel® also showed significant enhancement of antibody responses in animal models. [26]–[28] Therefore, we evaluated CpG ODN adjuvant in combination with AMA-C1/Alhydrogel in a phase 1 clinical trial.

## Methods

The protocol for this trial and supporting CONSORT checklist are available as supporting information; see Checklist S1 and Protocol S1.

### Study design

The study was conducted as a single-blind, randomized, dose escalating Phase 1 clinical trial in healthy volunteers, designed to evaluate the safety, reactogenicity, and immunogenicity of the AMA1-C1 vaccine formulated on Alhydrogel® and mixed with CPG 7909. The study was performed in three sequential cohorts, and escalation between cohorts did not occur until the previous cohort had received two vaccinations and the safety data from this group had been reviewed by a Safety Monitoring Committee.

The study was conducted under a protocol reviewed and approved by the Institutional Review Board (IRB) of the National Institute of Allergy and Infectious Disease (NIAID), and by the University of Rochester Research Subjects Review Board. The study was submitted to the U.S. Food and Drug Administration for review as part of Investigational New Drug application BB-IND#12029. The study was monitored for regulatory compliance and data quality by the Division of Microbiology and Infectious Diseases (DMID) of NIAID. Written informed consent was obtained from all volunteers prior to screening for eligibility for participation

### Participants

Participants were healthy volunteers between 18 and 45 years old recruited in the Rochester, New York area. Exclusion criteria included prior malaria infection, recent or planned travel to a malaria endemic country, and recent use of malaria prophylaxis. Because of the theoretical risk of inducing autoimmunity with CpG ODNs, potential subjects were screened for positive anti-double stranded DNA (ds-DNA), rheumatoid factor (RF), anti-nuclear antibody (ANA) and proteinuria. Volunteers also had normal results for screening laboratories (complete blood count, aspartate aminotransferase (AST), creatinine), no serologic evidence of hepatitis B or C infection, or antibody to human immunodeficiency virus. Urine pregnancy testing was performed at screening as well as prior to each vaccination for females.

### Interventions

AMA-1 antigens were expressed in the yeast *Pichia pastoris*, and 20 µg and 80 µg doses of AMA1-C1/Alhydrogel® were manufactured under cGMP conditions as previously described [14], [16]. cGMP CPG 7909 was supplied by Coley Pharmaceutical Group (Wellesley, MA) under a clinical trials agreement. CPG 7909 is a short synthetic ODN with a sequence of 5′-TCG TCG TTT TGT CGT TTT GTC GTT-3′ with all nucleotides linked with phosphorothioate bonds. CPG 7909 was supplied in sterile vials containing 1 mL at 10 mg/mL in hypertonic phosphate buffer at a pH 7.4. AMA1-C1/Alhydrogel+CPG 7909 was prepared as a point of injection formulation. Characterization and quality control were performed as previously described [27], [28]. Shortly before vaccination, 0.08 mL of CPG 7909 was drawn into a 0.30 mL (insulin) syringe and added to a single dose (0.7 mL) vial of AMA1-C1/Alhydrogel and gently mixed. The vaccine was administered no more than 6 hours after mixing and was kept at 4°C if not immediately administered. When ready to administer, 0.55 mL was withdrawn into a syringe, and the vaccine injected. Vaccine was given by intramuscular injection into the deltoid muscle on Days 0, 28, and 56, with successive vaccinations given in alternating arms. A 0.55 mL dose of AMA1-C1/Alhydrogel+CPG 7909 corresponds to either 20 µg or 80 µg of AMA1-C1 and 564 µg of CPG 7909.

Participants were sequentially enrolled and randomized within three dose escalating cohorts. In the first cohort, subjects were randomized to receive either 80 µg of AMA1-C1/Alhydrogel® (80 µg AMA1, n = 5), or 20 µg of AMA1-C1/Alhydrogel®+564 µg CPG 7909 (20 µg AMA1+CPG, n = 15). In the second cohort, subjects received either 80 µg AMA1/Alhydrogel® (n = 5) or 80 µg of AMA1-C1/Alhydrogel+564 µg CPG 7909 (80 µg AMA1+CPG, n = 15). In the third cohort, subjects received either 80 µg AMA1/Alhydrogel® (n = 20) or 80 µg AMA1/Alhydrogel®+564 µg CPG 7909 (n = 15). Randomization was performed using the enrolment module of the Internet Data Entry System (IDES), with the code list stored in a secured location at the study site. Participants remained blinded to their treatment assignment for the duration of the study.

### Outcomes

#### (i) Safety

Volunteers were observed for 30 minutes after each vaccination to evaluate for immediate adverse events, and were given diary cards to record events occurring during the first week after vaccination. Volunteers were seen in the study clinic at 3, 7, and 14 days after each vaccination, and at 28, 84, and 180 days after the third vaccine dose. Solicited adverse events included injection site pain, erythema, and induration, fever, headache, nausea, myalgia, arthralgia, and rash. Pain and solicited adverse events other than fever and urticaria were graded as follows: 0 = absent/none, 1 = easily tolerated, 2 = interferes with daily activity, 3 = prevents daily activity. Unless otherwise specified, non-solicited adverse events were graded as 0 = none, 1 = no effect on activities of daily living, 2 = partial limitation in activities of daily living, or treatment given, 3 = activities of daily living limited to <50% of baseline, or medical evaluation required. Hematological (Hb, WBC, and platelets) and biochemical (AST and creatinine) laboratory parameters were measured at screening and on days of immunization, and at 3 and 14 days after each vaccination; hematological parameters were also checked on Days 7. Anti-dsDNA, ANA, and RF were checked at screening, each day of vaccination, and at 7 days after each dose and 28 days after the third dose, and at the end of the study. All adverse events were graded for severity and relationship to study product. Adverse events which were unlikely, possibly, probably or definitely related to vaccination were considered to be associated with vaccination; adverse events definitely unrelated to vaccination were not associated. Serious adverse events (SAEs) were defined as any adverse event resulting in death, life threatening, requiring hospitalization, resulting in disability or incapacity or congenital anomaly or birth defect, or any other event which required intervention to prevent such outcomes.

#### (ii) Immunological Assays

Antibody responses to the AMA1 antigens were measured by enzyme linked immunosorbent assay (ELISA) on Days 0, 14, 28, 42, 56, 70, 84, 140 and 236. The standardized ELISA technique was performed as described previously [16], [17], [29]. A new human anti-AMA1 standard serum was made using a pool of plasma from 3 individuals receiving AMA1-C1/Alhydrogel+CPG 7909 vaccine in this study. The standard pool was assigned 460.9 Malaria Vaccine Development Branch (MVDB) ELISA units on AMA1-FVO and 578.0 units on AMA1-3D7. The AMA1 specific antibody concentration in the standard pool gave 0.0584 (SE±0.0012) µg/mL of AMA1-FVO-specific IgG for 1 ELISA unit, and 0.0473 (SE±0.0016) µg/mL of AMA1-3D7-specific IgG for 1 ELISA unit. The methods used to determine the concentration will be reported elsewhere (Miura et al, manuscript in preparation). Using these factors, all MVDB ELISA units were converted to µg/mL. Minimal detection level of this ELISA was 1.66 µg/mL and all values less than that were assigned as 0.83 µg/mL for statistical analysis.

In vitro parasite growth inhibition of homologous FVO and 3D7 *P.falciparium* strains was performed on sera from all volunteers on Days 0 and 70 using a standardized growth inhibition assay (GIA) as previously reported [16], [17]. GIA was performed using total IgG purified from individual sera and concentrated to 40 mg/mL. The final concentration of total IgG in the GIA wells was 10 mg/mL. The anti-AMA1 antibody concentrations (µg/mL) shown are the concentration of specific antibody in test wells. Heterologous in vitro parasite growth inhibition of L32 [13] and M24 [14] strains of parasites were performed. Due to the limitation of volume of purified IgGs, only ten total IgG samples with high growth-inhibitory activity to homologous parasites (≥68% inhibition to 3D7 parasites) were tested.

Samples were excluded from the ELISA and GIA statistical analysis for the time points after the first missed vaccination of volunteers who withdrew or were withdrawn from the study or if samples were unavailable.

### Statistical analysis

#### (i) Adverse Events

The frequency of adverse events stratified by dose cohort was summarized. Logistic regressions were used to investigate the effect of dose group on either local, systemic or laboratory adverse events. In each logistic regression, the response used for each subject was the most severe response observed, and all subjects were included regardless of withdrawal status. For the local or systemic reactions separate regressions were done for the odds of experiencing any reaction (mild, moderate, or severe vs. none) or higher severity reactions (moderate or severe vs. none or mild). When a dose group effect was found to be significant, pairwise tests using the same model were used to further investigate specific comparisons. No corrections were made for multiple comparisons. Statistical analysis for adverse events was performed by an independent data management group (EMMES Corporation) using SAS Version 9.1 (SAS Institute Inc., Cary, NC), and results were reviewed by the study statistician (MPF).

#### (ii) Antibody responses

To further delineate differences in antibody responses or in vitro parasite growth inhibitions between the 80 µg AMA1, 20 µg AMA1+CPG, and 80 µg AMA1+CPG groups, a Kruskal-Wallis test was performed for ELISA results with sera obtained on Days 42, and when found to be significant at the 0.05 level, was followed by a pairwise comparison using the Mann-Whitney U test at the 0.05 level with the associated Hodges-Lehmann confidence intervals. This procedure is known to bound the family-wise type I error at 0.05 [30]. We repeated the procedure for Day 70 ELISA and Day 70 GIA. To test for an accelerated CPG effect we compared the 80 µg AMA1 group to the 80 µg AMA1+CPG group by Mann-Whitney U test. For comparison of Day 42 and Day 70 AMA-FVO and AMA1-3D7 titers within the groups, a Wilcoxon signed rank test was used with Hodges-Lehmann confidence intervals. To determine significant differences between growth inhibition on the 4 parasite strains (3D7, FVO, L32, and M24), we used an all-subset procedure with Friedman's test, with the family-wise p-value defined as the lowest family-wise significance level that declares all pairs significantly different [30]. StatXact 8 Procs (Cytel Inc., Cambridge, MA, USA) was used for these analyses and *P* values of <0.05 were considered significant for both antibody and adverse event analyses.

To model the relationship between anti-AMA1 antibodies (X) and growth inhibition (Y), we used a Hill function:
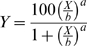
where *a* is the Hill coefficient, and *b* is the Ab_50_ (amount of antibody needed to give 50% growth inhibition). We fit the model by nonlinear least squares. We used an F test to see if calculating a separate Hill function for each vaccine group significantly improved the fit. Calculations were done in R 2.6.1 [31].

#### (iii) Sample size

The group size of 15 subjects for the 20 µg AMA1+CPG group, included for safety, gave a probability of about 0.8 for detecting one or more serious or severe AE that occur with a frequency of 0.1 per volunteer. The larger groups (80 µg AMA1 with and without CPG) were sized for a comparison of immunogenicity. Based on an analysis of the human antibody responses to a number of malaria antigens that had been previously tested in clinical trials, the observed coefficient of variation in the range of antibody concentrations was approximately 1.2–1.4. Assuming the larger coefficient of variation and assuming that one group had mean antibody concentrations that were at least 2.2 times the mean antibody concentrations of the other group, then the two-sided Mann-Whitney test assuming a significance of 0.05 performed on a sample size of 30 per group would have a power of greater than 0.80.

## Results

### Participant flow

**Figure 1:**
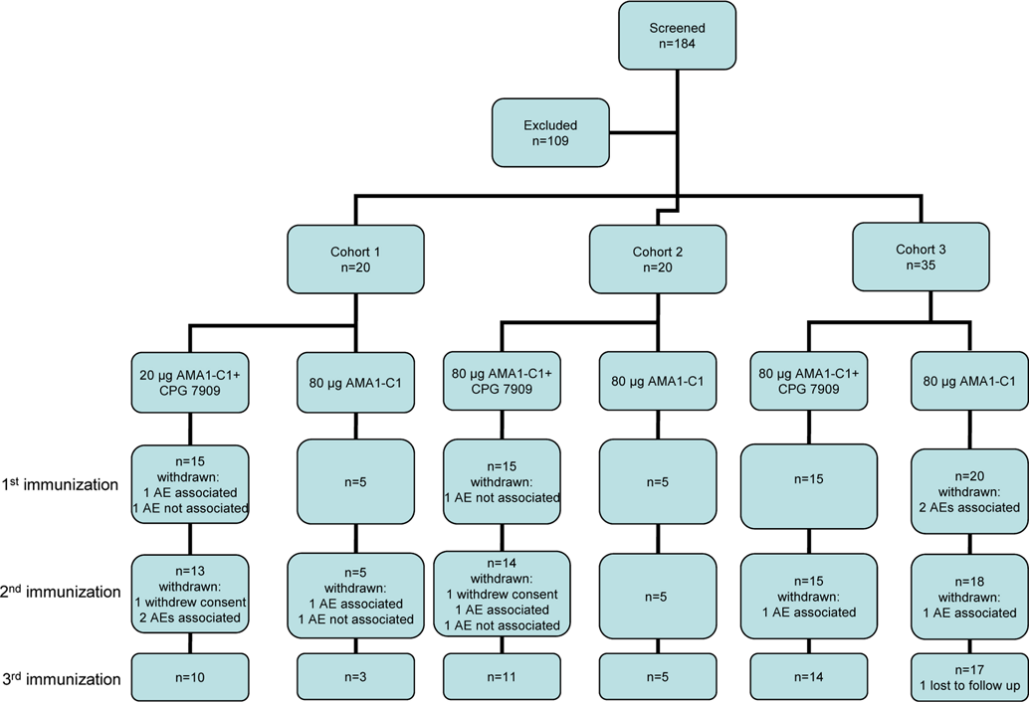
Participant Flow. Reasons for exclusion: Abnormal labs (48: 28 positive ANA, 9 positive RF, 1 positive anti-dsDNA, 10 other) Medical history (21) No show (19) Other (21).

The study was conducted at the University of Rochester School of Medicine from April 2005 to March 2007. 184 volunteers were screened to sequentially enroll 75 participants in three cohorts as described in Methods. The participant flow is shown in Figure 1. Fifteen participants did not complete the 3 dose vaccination series: two volunteers withdrew; 9 were withdrawn due to clinical or laboratory AEs associated with vaccination (unlikely, possibly, probably and definitely related), and 4 were withdrawn due to AEs not associated (definitely not related) with vaccination, including one volunteer who became pregnant as described below. Subjects who were withdrawn continued to be followed for adverse events, and in total 72/75 volunteers completed follow up to day 236.

### Safety and reactogenicity

Local reactogenicity and solicited systemic events are summarized in Table 1. Local reactions were mild or moderate, with the exception of one volunteer in the 80 µg AMA1 group who reported 150 mm induration at the injection site in a symptom diary starting on the day of the third vaccination and resolving 3 days later, and one volunteer in the 80 µg AMA1+CPG group who reported severe erythema 3 days after the second vaccination which lasted one day.

**Table 1:** Local and Solicited Systemic Reactogenicity.

20 µg+CPG		Vaccination 1				Vaccination 2				Vaccination 3			
		None N (%)	Mild N (%)	Moderate N (%)	Severe N (%)	None N (%)	Mild N (%)	Moderate N (%)	Severe N (%)	None N (%)	Mild N (%)	Moderate N (%)	Severe N (%)
**Systemic**	**Elevated Oral Temp**	15 (100)	0	0	0	9 (69)	3 (23)	0	0	10 (100)	0	0	0
	**Feverishness**	15 (100)	0	0	0	9 (69)	3 (23)	0	0	9 (90)	1 (10)	0	0
	**Fatigue**	12 (80)	3 (20)	0	0	5 (38)	4 (31)	3 (23)	0	9 (90)	1 (10)	0	0
	**Myalgia**	13 (87)	2 (13)	0	0	6 (46)	5 (38)	1 (8)	0	6 (60)	4 (40)	0	0
	**Headache**	9 (60)	5 (33)	1 (7)	0	6 (46)	4 (31)	2 (15)	0	7 (70)	2 (20)	1 (10)	0
	**Generalized Rash**	14 (93)	1 (7)	0	0	11 (85)	1 (8)	0	0	10 (100)	0	0	0
	**Loss of Appetite**	14 (93)	1 (7)	0	0	11 (85)	1 (8)	0	0	10 (100)	0	0	0
	**Any Systemic Symptom**	9 (60)	5 (33)	1 (7)	0	3 (23)	6 (46)	3 (23)	0	5 (50)	4 (40)	1 (10)	0
**Local**	**Pain**	5 (33)	9 (60)	1 (7)	0	3 (23)	8 (62)	1 (8)	0	2 (20)	7 (70)	1 (10)	0
	Erythema*	14 (93)	0	1 (7)	0	12 (92)	0	0	0	9 (90)	1 (10)	0	0
	Induration*	13 (87)	0	2 (13)	0	11 (85)	1 (8)	0	0	10 (100)	0	0	0
	**Any Local Symptom**	5 (33)	7 (47)	3 (20)	0	3 (23)	8 (62)	1 (8)	0	2 (20)	7 (70)	1 (10)	0
Any	**Any Symptom**	4 (27)	7 (47)	4 (27)	0	2 (15)	7 (54)	3 (23)	0	1 (10)	7 (70)	2 (20)	0
**80 µg**		**None N (%)**	**Mild N (%)**	**Moderate N (%)**	**Severe N (%)**	**None N (%)**	**Mild N (%)**	**Moderate N (%)**	**Severe N (%)**	**None N (%)**	**Mild N (%)**	**Moderate N (%)**	**Severe N (%)**
**Systemic**	**Elevated Oral Temp**	28 (93)	2 (7)	0	0	27 (96)	1 (4)	0	0	24 (96)	1 (4)	0	0
	**Feverishness**	29 (97)	1 (3)	0	0	27 (96)	0	1 (4)	0	24 (96)	1 (4)	0	0
	**Fatigue**	21 (70)	6 (20)	3 (10)	0	26 (93)	1 (4)	1 (4)	0	19 (76)	4 (16)	2 (8)	0
	**Myalgia**	26 (87)	3 (10)	1 (3)	0	26 (93)	2 (7)	0	0	23 (92)	1 (4)	1 (4)	0
	**Headache**	22 (73)	6 (20)	2 (7)	0	22 (79)	6 (21)	0	0	18 (72)	4 (16)	3 (12)	0
	**Generalized Rash**	30 (100)	0	0	0	28 (100)	0	0	0	24 (96)	1 (4)	0	0
	**Loss of Appetite**	28 (93)	2 (7)	0	0	26 (93)	1 (4)	1 (4)	0	23 (92)	1 (4)	1 (4)	0
	**Any Systemic Symptom**	14 (47)	11 (37)	5 (17)	0	20 (71)	7 (25)	1 (4)	0	15 (60)	6 (24)	4 (16)	0
**Local**	**Pain**	17 (57)	13 (43)	0	0	15 (54)	13 (46)	0	0	16 (64)	8 (32)	1 (4)	0
	Erythema*	26 (87)	4 (13)	0	0	24 (86)	3 (11)	1 (4)	0	21 (84)	4 (16)	0	0
	Induration*	29 (97)	1 (3)	0	0	27 (96)	1 (4)	0	0	21 (84)	3 (12)	0	1 (4)
	**Any Local Symptom**	14 (47)	16 (53)	0	0	15 (54)	12 (43)	1 (4)	0	13 (52)	10 (40)	1 (4)	1 (4)
**Any**	**Any Symptom**	7 (23)	18 (60)	5 (17)	0	11 (39)	15 (54)	2 (7)	0	9 (36)	11 (44)	4 (16)	1 (4)
**80 µg+CPG**		**None N (%)**	**Mild N (%)**	**Moderate N (%)**	**Severe N (%)**	**None N (%)**	**Mild N (%)**	**Moderate N (%)**	**Severe N (%)**	**None N (%)**	**Mild N (%)**	**Moderate N (%)**	**Severe N (%)**
**Systemic**	**Elevated Oral Temp**	28 (93)	1 (3)	1 (3)	0	28 (97)	1 (3)	0	0	23 (92)	1 (4)	1 (4)	0
	**Feverishness**	25 (83)	0	5 (17)	0	24 (83)	4 (14)	1 (3)	0	21 (84)	3 (12)	1 (4)	0
	**Fatigue**	18 (60)	4 (13)	7 (23)	1 (3)	21 (72)	7 (24)	1 (3)	0	20 (80)	4 (16)	0	1 (4)
	**Myalgia**	20 (67)	4 (13)	6 (20)	0	19 (66)	7 (24)	2 (7)	1 (3)	18 (72)	4 (16)	3 (12)	0
	**Headache**	16 (53)	5 (17)	9 (30)	0	20 (69)	6 (21)	3 (10)	0	17 (68)	6 (24)	2 (8)	0
	**Generalized Rash**	28 (93)	2 (7)	0	0	28 (97)	1 (3)	0	0	25 (100)	0	0	0
	**Loss of Appetite**	25 (83)	3 (10)	2 (7)	0	28 (97)	0	0	1 (3)	23 (92)	1 (4)	1 (4)	0
	**Any Systemic Symptom**	10 (33)	5 (17)	14 (47)	1 (3)	14 (48)	11 (38)	3 (10)	1 (3)	16 (64)	5 (20)	3 (12)	1 (4)
**Local**	**Pain**	10 (33)	13 (43)	7 (23)	0	4 (14)	21 (72)	4 (14)	0	10 (40)	12 (48)	3 (12)	0
	Erythema*	27 (90)	1 (3)	2 (7)	0	23 (79)	1 (3)	4 (14)	1 (3)	23 (92)	1 (4)	1 (4)	0
	Induration*	27 (90)	2 (7)	1 (3)	0	24 (83)	1 (3)	4 (14)	0	23 (92)	1 (4)	1 (4)	0
	**Any Local Symptom**	10 (33)	11 (37)	9 (30)	0	3 (10)	18 (62)	7 (24)	1 (3)	10 (40)	10 (40)	5 (20)	0
**Any**	**Any Symptom**	5 (17)	8 (27)	16 (53)	1 (3)	2 (7)	16 (55)	9 (31)	2 (7)	7 (28)	11 (44)	6 (24)	1 (4)

Local reactions were significantly more likely in the 80 µg AMA1+CPG group versus the 80 µg AMA1 group (OR 14.50, 95% CI 1.72–122.40, p = 0.0140), and reactions were more likely to be of a higher severity (moderate or severe versus mild or none) (OR 15.54, 95% CI 3.81–63.36, p = 0.0001). Combining the CPG groups also showed a higher likelihood of a local reaction (OR 20.00, 95% CI 1.75, 480.26, p = 0.019), and a higher likelihood of a more severe local reaction (OR 50.88, 95% CI 3.26–794.32, p = 0.0051) versus the group which did not receive CPG. When testing for an overall dose group effect on solicited systemic events, there was no significant difference when measuring any events (p = 0.546), but there was a significant difference for higher severity events (p = 0.024). Volunteers in the 80 µg AMA1+CPG group were significantly more likely to experience a higher severity event compared to the 80 µg AMA1 group (OR 4.57, 95% CI 1.45–14.39, p = 0.0094). Combining the CPG groups also showed a higher probability of a more severe systemic event in comparison to the non-CPG group (OR 9.14, 95% CI 1.01, 83.04, p = 0.0493).

Three volunteers were withdrawn due to rash related to vaccination. One volunteer receiving 20 µg AMA1+CPG developed a grade 1 papular non-pruritic rash on his chest, back, and upper arms 7 days after receiving his second vaccination. A dermatologic consultation was obtained and folliculitis/acne possibly related to vaccination was diagnosed. The rash lasted 45 days and he was not revaccinated. One volunteer receiving 80 µg AMA1+CPG developed a grade 1 erythematous, papular, non-pruritic rash on her chest and back 5 days after first vaccination. The rash had substantially subsided by day 28 and the volunteer received a second vaccination on Day 28. On Day 31 there were increased macules in the same distribution and a decision was made not to administer the third vaccination. The rash was completely resolved by Day 58. The third volunteer developed a grade 2 rash 17 days after receiving his first vaccination with 80 µg AMA1; this was a fine papular rash on the trunk, arms, and legs associated with itchy eyes and rhinitis. Tests for mononucleosis and streptococcal infection done by the primary physician were negative and the rash resolved after 4 days. Another volunteer was withdrawn due to Grade 3 epigastric pain after second vaccination with 80 µg AMA1+CPG. Other Grade 3 AEs judged to be possibly related to vaccination included vomiting, loss of appetite, body aches, and fatigue. All Grade 3 systemic AEs resolved within 3 days.

Three volunteers were withdrawn due to a positive ANA. One volunteer who received 20 µg AMA1+CPG was positive (1∶80) on Day 0 and continued to have low level fluctuations until the end of the study; RF and anti-dsDNA remained normal and this was considered not associated with vaccination. ANA elevations in the other two volunteers were considered associated with vaccination. One volunteer received 80 µg AMA1 without CPG; this volunteer had a mild elevation (1∶80) on the day of second vaccination which returned to normal 6 weeks later; RF and anti-dsDNA remained normal throughout. One volunteer who also received 80 µg AMA1 without CPG had a normal ANA at baseline, with a mild elevation (1∶80) 7 days after vaccination and 1∶1280 at second vaccination; this volunteer also had intermittently positive RF. After consultation with the safety monitoring committee, the protocol was amended to allow continued participation if ANA was positive at vaccination, as long as anti-dsDNA and proteinuria were both negative. Subsequently, four volunteers had ANAs of 1∶80 either at day of first vaccination, day of second vaccination, or early in their participation; all received three doses of vaccine as per protocol and all returned to normal, with some low level fluctuations. Three of these four volunteers received vaccine with CPG. One volunteer was withdrawn due to positive anti-dsDNA (82 IU, normal 0–70) 7 days after first vaccination with 20 µg AMA1+CPG; ANA and RF remained negative throughout and anti-dsDNA was negative when rechecked 21 days later. No volunteers had clinical evidence of autoimmune disease. The other two withdrawals associated with vaccination were due to low hemoglobin in a volunteer who received two doses of 20 µg AMA1+CPG, and one volunteer who received one dose of 80 µg AMA1 who experienced a worsening of pre-existing chest pain related to anxiety.

Laboratory adverse events are summarized in Table 2. There are no significant differences between study groups for any parameter. Transient decreases in white blood cells and platelets are an expected observation with administration of CPG 7909 [32]. In this study there were no adverse events as a result of decreases in platelets. Most decreases in WBCs were mild (Grade 1), with 1 volunteer in the 20 µg AMA1+CPG group and one in the 80 µg AMA1 group experiencing a Grade 2 decrease. Several volunteers distributed among the dose groups had decreases in hemoglobin, thought by the PI and SMC to be related to the volume of blood drawn during the study (a total of 770 mL, with 655 mL drawn during the first 90 days). Several volunteers in both the CPG and non CPG groups had mild elevations of RF (10 to 40 IU); all returned to normal (<10 IU) within 14 days except for one volunteer who received 80 µg AMA1+CPG and had a persistent titer of 10 IU at the end of the study with no other laboratory or clinical abnormalities.

**Table 2:** Percentage of Subjects with Abnormal Laboratory Results.

Parameter	Study Group		
	20 µg+CPG 7909	80 µg	80 µg+CPG 7909
	(N = 15)	(N = 30)	(N = 30)
	%	%	%
Elevated AST	13.3	10.0	0
Elevated Creatinine	0	3.3	3.3
Decreased hemoglobin	26.7	6.7	10.0
Decreased platelets	0	0	0
Decreased WBC	6.7	30.0	30.0
ANA	6.7	13.3	16.7
Anti-ds DNA	6.7	0	0
Rheumatoid Factor	6.7	3.3	20.0
Proteinuria	6.7	6.7	16.7

### Serious adverse events

Five SAEs were reported in this study. All were assessed by the investigators as either unrelated (situational depression, rib fracture, testicular cancer, recurrence of bipolar disorder) or unlikely related (pregnancy with miscarriage) to study treatment or participation. Of note, one volunteer was found to be pregnant at the visit scheduled for her third vaccination, which she did not receive. Viability of the pregnancy was not confirmed by ultrasound or serial HCG and she subsequently had a spontaneous miscarriage. This event was judged by the investigators to be unlikely related to vaccination due to the volunteer's history of polycystic ovarian disease, which is associated with early pregnancy loss [33].

### Immune responses to AMA1

#### (i) Anti-AMA1 IgG antibodies

**Figure 2:**
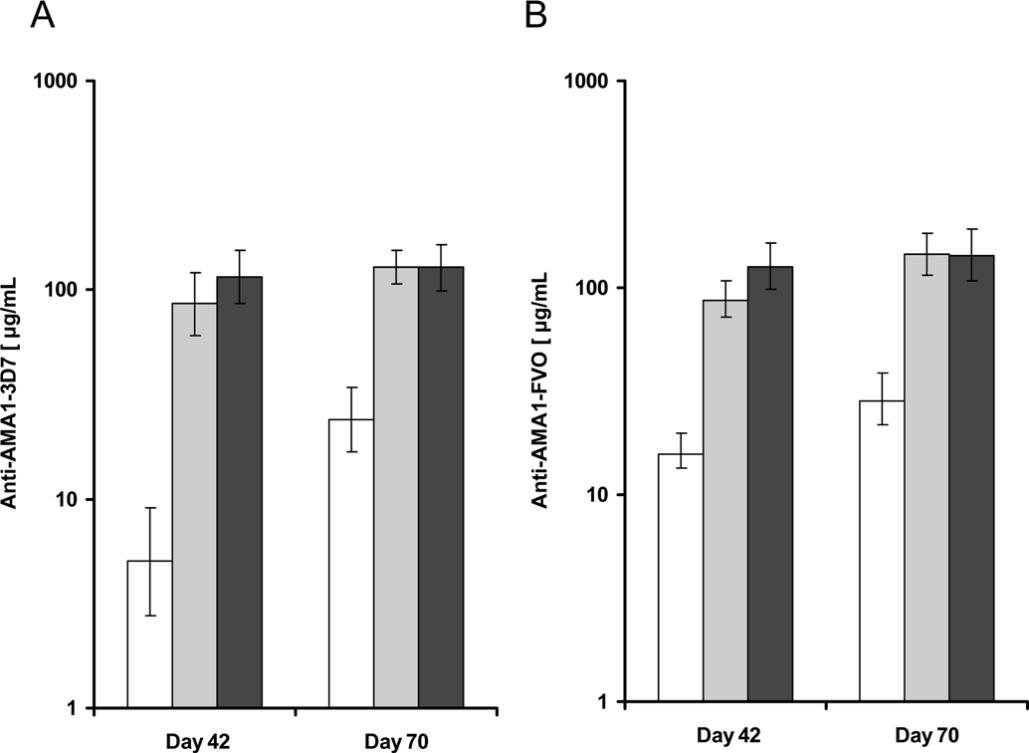
Anti-AMA1 antibody responses: Day 42 and 70 AMA1 antibody responses in recipients of the 20 or 80 µg AMA1-C1/Alhydrogel±CPG 7909 vaccines. Antibody responses against AMA1-3D7 (A) and AMA1-FVO (B) measured by ELISA in sera collected on Day 42 (14 days postvaccination 2) and Day 70 (14 days postvaccination 3). ELISA results are shown as the geometric mean of AMA1-specific antibody (µg/mL) with standard error. The bars represents 80 µg AMA1 (white bar, *n* = 25), 20 µg AMA1+CPG (grey bar, *n* = 12), and 80 µg AMA1+CPG (black bar, *n* = 29).

**Figure 3:**
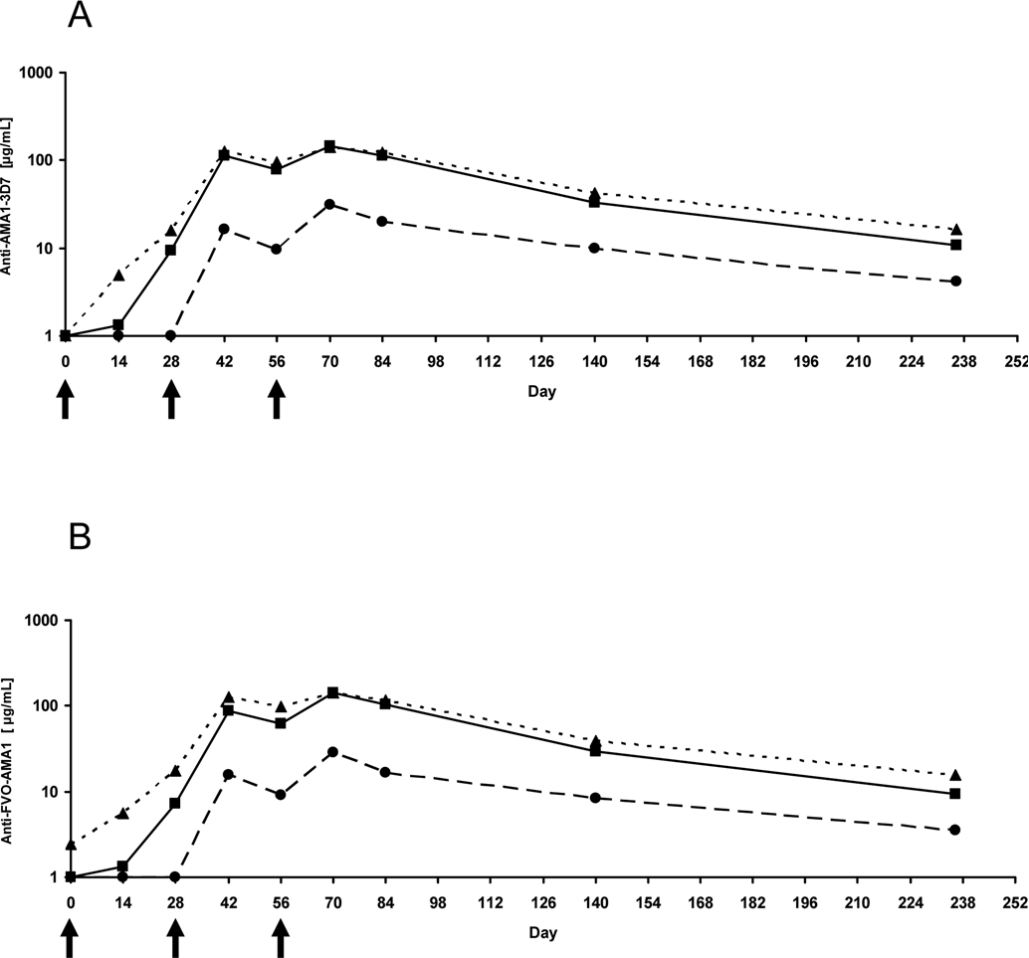
Longevity of antibody responses: AMA1 antibody responses in recipients of the 20 or 80 µg AMA1-C1/Alhydrogel±CPG 7909 vaccines. Antibody responses against AMA1-3D7 (A) and AMA1-FVO (B) measured by ELISA in sera collected on Day 0 through to Day 236. ELISA results are shown as the geometric mean of AMA1-specific antibody (µg/mL). The lines represents 80 µg AMA1 (black circles/dashed line), 20 µg AMA1+CPG (black squares/solid line), and 80 µg AMA1+CPG (black triangles/dotted line). Arrows indicate the days of vaccination.

Antibody levels against the constituent AMA1 alleles in the vaccines were measured by ELISA at different time points following vaccination. Antibody responses to AMA1-3D7 and AMA1-FVO were measured separately to study the contribution of each allele to the overall antibody response. Results for FVO were similar to those for 3D7 and are not presented in the text (see Figure 2B and Figure 3B for FVO results).

The protocol anticipated maximum antibody responses on Day 70, 14 days after third vaccination; however, the increase in antibody responses from Day 42 to Day 70 was significant only for the 80 µg AMA1 group, as shown by the estimates of fold change with 95% confidence intervals and p-values. The difference between the Day 42 and Day 70 results for the anti-AMA1-3D7 responses for each group are: 80 µg AMA1 (2.37 fold change, 95% CI 1.53–3.67, p = 0.0001), 20 µg AMA1+CPG (1.17 fold change, 95% CI 0.81–2.06, p = 0.432), and 80 µg AMA1+CPG (1.12 fold change, 95% CI 0.99–1.26, p = 0.075).

Kruskal-Wallis tests showed significant differences between 80 µg AMA1, 20 µg AMA1+CPG and 80 µg AMA1+CPG groups for Day 42 and 70 anti-AMA1-3D7 responses derived from individual sera (all p<0.0001, Figure 2). Pair-wise comparisons of the anti-AMA1-3D7 responses between the groups revealed that on Day 42 the 20 µg AMA1+CPG or 80 µg AMA1+CPG antibody responses were respectively 11.57 (95% CI 5.72–25.98) and 14.24 (95% CI 9.22–26.60) fold higher than those observed with the 80 µg AMA1 group and that the differences were significant (for both cases *P*<0.0001). However, the 80 µg AMA1+CPG group was not significantly higher (fold change of 1.15, 95% CI 0.78–1.81, p = .4194) than the 20 µg AMA1+CPG group on Day 42.

Comparisons of the antibody responses between the groups revealed that on Day 70 the 20 µg AMA1+CPG or 80 µg AMA1+CPG responses were respectively 5.94 (95% CI 3.22–9.15) and 5.50 (95% CI 3.80–7.91) fold higher than those observed with the 80 µg AMA1 group for AMA1-3D7 (for both cases *P*<0.0001). The 80 µg AMA1+CPG group was not significantly higher (fold change of 1.01 95% 0.68–1.39, p = .9220) than the 20 µg AMA1+CPG group on Day 70.

The addition of CPG 7909 to 80 µg AMA1 accelerated the anti-AMA1 antibody production. The 80 µg AMA1+CPG group was significantly higher than the 80 µg AMA1 group at both Day 14 and Day 28 (p<0.0001 for both).

Longevity of antibody responses was investigated by plotting the mean Day 0 to Day 236 anti-AMA1-3D7 and anti-AMA1-FVO responses derived from individual sera for 80 µg AMA1, 20 µg AMA1+CPG and 80 µg AMA1+CPG groups (Figure 3). Antibody levels on day 236, the last day of the study, declined from their peak on Day 70. At Day 236, the 20 µg AMA1+CPG and 80 µg AMA1+CPG groups were still significantly higher than the 80 µg AMA1 group for 3D7, with fold changes of 2.90 (95% CI 1.73–5.10, p<0.0001) and 4.65 (95% CI 3.14–7.20, p<0.0001), respectively. At Day 236 there is a small but significant increase of the 80 µg AMA1+CPG group over the 20 µg AMA1+CPG group (fold change of 1.61, 95% CI 1.06–2.13, p = 0.021).

All of the above statistical analyses give similar results for both anti-AMA1 3D7 and FVO responses.

#### (ii) In vitro growth inhibition assay

**Figure 4:**
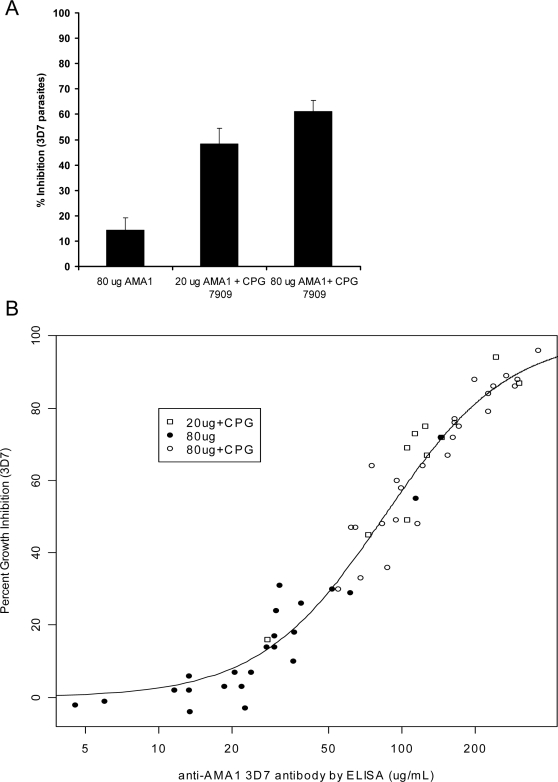
Functional Immunological response: Homologous in vitro parasite growth inhibition in recipients of the 20 or 80 µg AMA1-C1/Alhydrogel±CPG 7909 vaccines. Day 70 in vitro parasite growth inhibition response against 3D7 parasites measured by GIA using total IgG purified from individual sera. (A) Arithmetic mean of % inhibition against 3D7 parasites is shown with standard error bars. (B) The concentration of Day 70 AMA1-specific antibody (µg/mL) to test wells (x-axis) is plotted against % inhibition (y-axis). The individual data points represent 80 µg AMA1 (black circles, *n* = 23), 20 µg AMA1+CPG (white squares, *n* = 10), and 80 µg AMA1+CPG (white circles, *n* = 25).

To determine whether antibodies elicited by immunization had functional activity against homologous *P. falciparum* parasites, we tested the abilities of Day 70 sera to inhibit in vitro growth of the *P. falciparum* 3D7 and FVO parasite strains. To minimize the possibility of non-specific in vitro inhibition of parasite growth and to ensure that the inhibitory activity was attributable to the antibodies alone, we used IgG purified from each serum sample (Figure 4). Substantial inhibition of both *P. falciparum* 3D7 and FVO parasite growth was achieved for the 20 or 80 µg AMA1+CPG groups with inhibitions ranging from 16 to 96% for 3D7 (arithmetic means 65 and 66%, respectively) and 5–96% for FVO (arithmetic means 40 and 44%). For the 80 µg AMA1 group, the in vitro growth inhibitions ranged from −4 to 72% for 3D7 (arithmetic mean 16%) and −8 to 58% for FVO (arithmetic mean 6%); prevaccination IgG showed <1% mean inhibition. There was a significant vaccine group effect overall for both 3D7 and FVO (p<0.0001 for both). Both CPG groups had significantly higher inhibition than the 80 µg AMA1 group; the 20 µg AMA1+CPG group had a higher inhibition than the 80 µg AMA1 group by 54.5 percentage points for 3D7 (95% CI 38–69, p<0.0001) and 29 for FVO (95% CI 19–41, p<0.0001), and the 80 µg AMA1+CPG group had a higher inhibition than the 80 µg AMA1 group by 53 percentage points for 3D7 (95% CI 41–65, p<0.0001) and 36 for FVO (95% CI 29–47, p<0.0001). There were no significant differences between the 20 µg and 80 µg AMA1+CPG groups, although the 80 µg AMA1+CPG group had non-significantly higher inhibition: for 3D7 it was higher by 0.5 percentage points (95% CI −17–16, p = 0.950) for FVO it was higher by 7 percentage points (95% CI −6–23, p = 0.353). The Day 42 data give similar values (results not shown). IgG samples with the highest growth-inhibitory activity were tested for specificity of inhibition by reversal of the GIA using specific antigen. Inclusion of AMA1 antigen in the GIA completely reversed the inhibition seen (data not shown), establishing the specificity of the biological activity.

Although there was a significant difference of growth-inhibitory activity between groups with and without CPG, it was not clear whether the difference in activity was caused only by differences in amount of antibody between the groups or whether antibody elicited by the vaccines containing CPG had greater biologic activity. Therefore the individual Day 70 data against 3D7 parasite was plotted as shown in Figure 4B. When all of the data points were included, the Hill regression model of the relationship between anti-AMA1 3D7 antibodies and the homologous growth inhibition had a good fit. The Hill coefficient was 1.70 (95% CI 1.53–1.89). The antibody level at 50% growth inhibition (Ab_50_) was 84.4 µg/mL (95% CI 79.3–89.7). The model that used a separate Hill function for each vaccine group did not create a significantly better fit (p = 0.476), so the data do not show any significant difference in biological activity Similar results were obtained for the FVO parasites (data not shown).

**Figure 5:**
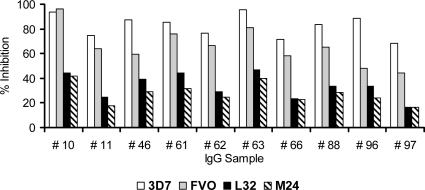
Functional Immunological response: Heterologous in vitro parasite growth inhibition. Ten available samples on Day 70 with high growth-inhibitory activity to homologous parasites (≥68% inhibition to 3D7 parasites) were selected. Volunteers 10, 11 and 46 received 20 µg AMA1+CPG; Volunteers 61, 62, 63, 88, 96 and 97 received 80 µg AMA1+CPG 7909 and Volunteer 66 received 80 µg AMA1. Day 70 in vitro parasite growth inhibition response against 3D7 parasites (white bar), FVO (grey bar) and L32 (black bar) and M24 (hashed bar) measured by GIA using total IgG (10 mg/mL in GIA well) purified from individual sera.

To determine whether antibodies elicited by immunization had biologic activity against heterologous *P. falciparum* parasites, we tested the activity of Day 70 purified total IgG. Due to the limitation in volumes of purified IgGs, ten total IgG samples with high growth-inhibitory activity to homologous parasites (≥68% inhibition to 3D7 parasites) were tested with *P. falciparum* L32 and M24 parasite strains and compared to the inhibition on the homologous 3D7 and FVO strains (Figure 5). The average inhibition on 3D7, FVO, L32, and M24 parasites was 83%, 66%, 34%, and 28%, respectively. There were significant differences in inhibition between all pairs of parasite strains with the ordering 3D7>FVO>L32>M24 (i.e., L32>M24, FVO>M24, 3D7>M24, etc. family-wise p = 0.043).

## Discussion

Local adverse reactions were more frequent and local and systemic adverse events were significantly more likely to be more severe when CPG 7909 was added to the AMA1-C1/Alhydrogel vaccine. This has been seen in some previous studies using a combination of CPG 7909 with licensed vaccines [22]–[24] and was not unexpected. As with any vaccine, risk must be weighed against benefit. For a vaccine intended to prevent severe malaria, the risk of greater reactogenicity may be outweighed by enhanced immunogenicity. Most adverse reactions were mild or moderate, severe reactions were of short duration, and no serious adverse events judged possibly, probably, or definitely related to vaccination occurred. This safety profile was judged to be acceptable given the greatly enhanced immunogenicity of the AMA1-C1/Alhydrogel+CPG 7909 vaccine, and further development of this blood-stage vaccine in malaria endemic areas.

There is a theoretical risk of inducing autoimmune disease with CpG ODN, and markers for this were monitored frequently in this study. One volunteer who received CPG was withdrawn from further vaccinations after developing a transiently low positive anti-dsDNA which subsequently returned to normal. Other studies of CPG 7909 have also rarely seen transiently elevated anti-dsDNA after vaccination [22]; no clinical events related to this have been reported in studies using CPG 7909 in a vaccine to date. ANA and RF are relatively non-specific tests of limited usefulness in low risk populations [34], [35]. In this study this is illustrated by the high number of positive results before vaccination, and frequent fluctuations from baseline in both the CPG and non-CPG groups (Table 2). Three volunteers with baseline or early elevations of ANA received three doses of vaccine containing CPG without an increase in ANA titer; the volunteer who had a higher titer ANA (1∶1280) did not receive CPG. Less frequent monitoring using the more specific of these markers, i.e., monitoring anti-dsDNA alone, and checking ANA and RF if anti-dsDNA is positive or if clinical symptoms are present, is likely to be sufficient for future studies.

While assessment of the safety of the vaccine formulation was the primary goal of this study, an important secondary objective was to determine the ability of the AMA1-C1/Alhydrogel+CPG 7909 vaccine to elicit humoral immune responses in malaria-naïve individuals after primary vaccination and the ability to boost memory responses with revaccination. While there was virtually no detectable antibody response to either AMA1-FVO or AMA1-3D7 after the first dose of 80 µg AMA1 on Day 28 in malaria-naïve volunteers, an antibody response was detected in volunteers who received either 20 or 80 µg of AMA1+CPG. This dramatic increase in anti-AMA1 antibody concentrations continued to be significant post second and third immunizations on Days 42 and 70 and to the end of the study on Day 236. The difference in antibody concentration after the second and third immunizations for the 80 µg AMA1+CPG group was small (1.12 fold) and non-significant, indicating that two immunizations were sufficient to elicit a near-maximal antibody response at this dose level. Two weeks after the three immunizations there was also no significant difference in AMA1 antibody concentration between 20 µg and 80 µg of AMA1-C1+CPG. Thus the addition of CPG may be “dose sparing”, as has been observed with other CPG vaccine clinical studies [22], [23].

While there is considerable experimental support for the choice of AMA1 as a vaccine candidate, the selection of this antigen is potentially complicated by the fact that it displays significant amino acid variability in different field isolates. At least 64 amino acid residues have been shown to have one or more substitutions, but the impact of this polymorphism with respect to protective responses is unknown [10]. To address this issue, the AMA1-C1 vaccine was designed to include AMA1 proteins based on the sequences of two relatively diverse *P. falciparum* strains: FVO and 3D7. The AMA1 sequences from these two parasite strains differ by 25 amino acids. By incorporating both the FVO and 3D7 alleles into the AMA1-C1 vaccine, the goal was to induce a broader immune response than could be achieved by vaccination with either individual component alone. The anti-AMA1 antibodies induced by the AMA1-C1 vaccine recognize the native parasite protein and inhibit growth of the FVO and 3D7 strains of *P. falciparum* in vitro. The addition of CPG 7909 to the AMA1-C1/Alhydrogel vaccine in humans elicited AMA1 specific immune IgG that dramatically increased the growth inhibition to levels as high as 96%. This growth-inhibitory activity was significantly better than that from AMA1-C1/Alhydrogel in this paper as well as what has been previously reported [16]. However, all GIA data points could be plotted on the same curve regardless of formulation, suggesting that the intrinsic functional activity of the antibodies was not different with and without the addition of CPG 7909.

Having converted the ELISA units to µg/mL and using a widely accepted standardized GIA, it is now possible to develop a standardized inhibitory constant such as the concentration of AMA1 antibody required to give 50% inhibition of homologous parasite growth (i.e. Ab_50_). The Ab_50_ could be used to compare GIA results between blood-stage vaccine trials. However, it should be noted that the correlation of growth-inhibitory activity with in vivo reduction in parasite growth or protection in field trials against clinical malaria remains to be established.

The growth inhibition of these same total IgGs on the heterologous parasite strains L32 and M24 was not as high and gave rise to levels approximately half or less of those observed for the homologous FVO and 3D7 parasite strains. While heterologous inhibition was significantly decreased compared to homologous inhibition, some cross-strain inhibition was demonstrated. To potentially further broaden protection, MVDB is developing a second generation vaccine which will combine a third, more divergent AMA1 allele with AMA1-C1 and which will be evaluated in humans in the near future. Other approaches include developing an AMA1 vaccine that incorporates a combination of synthetic Diversity-Covering PfAMA1 sequences to provide a broader functional immune response[15].

In conclusion, in this Phase 1 trial the AMA1-C1/Alhydrogel+CPG 7909 vaccine demonstrated an adequate safety profile and was highly immunogenic in malaria-naïve individuals. Additionally, the functional properties of these antibodies were demonstrated by their ability to inhibit parasite growth in vitro. AMA1-C1/Alhydrogel+CPG 7909 is currently under evaluation in a Phase 1 endemic adult trial and if found to be safe a Phase 1 pediatric trial is planned. This would be the first use of CPG 7909 in a pediatric population for any indication.

## Supporting Information

Checklist S1 Consort Checklist.
(0.11 MB PDF)

Protocol S1 Trial Protocol.
(0.63 MB PDF)

## References

[1] WHO (2005). World Malaria Report, World Health Organization..

[2] Anders RF, Crewther PE, Edwards S, Margetts M, Matthew ML (1998). Immunisation with recombinant AMA-1 protects mice against infection with Plasmodium chabaudi.. Vaccine.

[3] Narum DL, Ogun SA, Thomas AW, Holder AA (2000). Immunization with parasite-derived apical membrane antigen 1 or passive immunization with a specific monoclonal antibody protects BALB/c mice against lethal Plasmodium yoelii yoelii YM blood-stage infection.. Infect Immun.

[4] Deans JA, Knight AM, Jean WC, Waters AP, Cohen S (1988). Vaccination trials in rhesus monkeys with a minor, invariant, Plasmodium knowlesi 66 kD merozoite antigen.. Parasite Immunol.

[5] Collins WE, Pye D, Crewther PE, Vandenberg KL, Galland GG (1994). Protective immunity induced in squirrel monkeys with recombinant apical membrane antigen-1 of Plasmodium fragile.. Am J Trop Med Hyg.

[6] Stowers AW, Kennedy MC, Keegan BP, Saul A, Long CA (2002). Vaccination of monkeys with recombinant Plasmodium falciparum apical membrane antigen 1 confers protection against blood-stage malaria.. Infect Immun.

[7] Marshall VM, Zhang L, Anders RF, Coppel RL (1996). Diversity of the vaccine candidate AMA-1 of Plasmodium falciparum.. Mol Biochem Parasitol.

[8] Polley SD, Conway DJ (2001). Strong diversifying selection on domains of the Plasmodium falciparum apical membrane antigen 1 gene.. Genetics.

[9] Escalante AA, Grebert HM, Chaiyaroj SC, Magris M, Biswas S (2001). Polymorphism in the gene encoding the apical membrane antigen-1 (AMA-1) of Plasmodium falciparum. X. Asembo Bay Cohort Project.. Mol Biochem Parasitol.

[10] Healer J, Crawford S, Ralph S, McFadden G, Cowman AF (2002). Independent translocation of two micronemal proteins in developing Plasmodium falciparum merozoites.. Infect Immun.

[11] Remarque EJ, Faber BW, Kocken CH, Thomas AW (2008). Apical membrane antigen 1: a malaria vaccine candidate in review.. Trends Parasitol.

[12] Dutta S, Lee SY, Batchelor AH, Lanar DE (2007). Structural basis of antigenic escape of a malaria vaccine candidate.. Proc Natl Acad Sci U S A.

[13] Duan S, Mu J, Thera M, Joy D, Kosakovsky Pond S (2008). Population structure of the genes encoding the polymorphic P. falciparum apical membrane antigen1: implications for vaccine design.. Proc Natl Acad Sci U S A.

[14] Kennedy MC, Wang J, Zhang Y, Miles AP, Chitsaz F (2002). In vitro studies with recombinant Plasmodium falciparum apical membrane antigen 1 (AMA1): production and activity of an AMA1 vaccine and generation of a multiallelic response.. Infect Immun.

[15] Remarque EJ, Faber BW, Kocken CH, Thomas AW (2008). A diversity-covering approach to immunisation with Plasmodium falciparum AMA1 induces broader allelic recognition and growth inhibition responses in rabbits.. Infect Immun.

[16] Malkin EM, Diemert DJ, McArthur JH, Perreault JR, Miles AP (2005). Phase 1 clinical trial of apical membrane antigen 1: an asexual blood-stage vaccine for Plasmodium falciparum malaria.. Infect Immun.

[17] Dicko A, Diemert DJ, Sagara I, Sogoba M, Niambele MB (2007). Impact of a Plasmodium falciparum AMA1 Vaccine on Antibody Responses in Adult Malians.. PLoS ONE.

[18] Dicko A, Sagara I, Ellis R, Miura K, Guindo O (2008). Phase 1 Study of a Combination AMA1 Blood Stage Malaria Vaccine in Malian Children.. PLoS ONE.

[19] ClinicalTrials.gov (2006). http://www.ClinicalTrials.gov.

[20] Epstein JE, Giersing B, Mullen G, Moorthy V, Richie TL (2007). Malaria vaccines: are we getting closer?. Curr Opin Mol Ther.

[21] Girard MP, Reed ZH, Friede M, Kieny MP (2007). A review of human vaccine research and development: malaria.. Vaccine.

[22] Cooper CL, Davis HL, Angel JB, Morris ML, Elfer SM (2005). CPG 7909 adjuvant improves hepatitis B virus vaccine seroprotection in antiretroviral-treated HIV-infected adults.. Aids.

[23] Cooper CL, Davis HL, Morris ML, Efler SM, Adhami MA (2004). CPG 7909, an immunostimulatory TLR9 agonist oligodeoxynucleotide, as adjuvant to Engerix-B HBV vaccine in healthy adults: a double-blind phase I/II study.. J Clin Immunol.

[24] Cooper CL, Davis HL, Morris ML, Efler SM, Krieg AM (2004). Safety and immunogenicity of CPG 7909 injection as an adjuvant to Fluarix influenza vaccine.. Vaccine.

[25] Klinman DM (2006). CpG oligonucleotides accelerate and boost the immune response elicited by AVA, the licensed anthrax vaccine.. Expert Rev Vaccines.

[26] Mullen GE, Giersing BK, Ajose-Popoola O, Davis HL, Kothe C (2006). Enhancement of functional antibody responses to AMA1-C1/Alhydrogel, a Plasmodium falciparum malaria vaccine, with CpG oligodeoxynucleotide.. Vaccine.

[27] Mullen GE, Aebig JA, Dobrescu G, Rausch K, Lambert L (2007). Enhanced antibody production in mice to the malaria antigen AMA1 by CPG 7909 requires physical association of CpG and antigen.. Vaccine.

[28] Aebig JA, Mullen GE, Dobrescu G, Rausch K, Lambert L (2007). Formulation of vaccines containing CpG oligonucleotides and alum.. J Immunol Methods.

[29] Miura K, Orcutt AC, Muratova OV, Miller LH, Saul A (2008). Development and characterization of a standardized ELISA including a reference serum on each plate to detect antibodies induced by experimental malaria vaccines.. Vaccine.

[30] Campbell G, Skillings J (1985). Nonparametric stepwise multiple comparison procedures.. Journal of the American Statistical Association.

[31] Team RDC (2007). R: A language and environment for statistical computing.

[32] Krieg AM, Efler SM, Wittpoth M, Al Adhami MJ, Davis HL (2004). Induction of systemic TH1-like innate immunity in normal volunteers following subcutaneous but not intravenous administration of CPG 7909, a synthetic B-class CpG oligodeoxynucleotide TLR9 agonist.. J Immunother.

[33] van der Spuy ZM, Dyer SJ (2004). The pathogenesis of infertility and early pregnancy loss in polycystic ovary syndrome.. Best Pract Res Clin Obstet Gynaecol.

[34] Shmerling RH, Delbanco TL (1991). The rheumatoid factor: an analysis of clinical utility.. Am J Med.

[35] Keren DF (2002). Antinuclear antibody testing.. Clin Lab Med.

