# Supplementary material for: Phase 1 Trial of AMA1-C1/Alhydrogel plus CPG 7909: An Asexual Blood-Stage Vaccine for Plasmodium falciparum Malaria
Source: PLoS One. 2008 Aug 13;3(8):e2940. doi: 10.1371/journal.pone.0002940 (PMC2491586; doi:10.1371/journal.pone.0002940)
Supplement: Protocol S1 — Trial Protocol. (0.63 MB PDF) [file pone.0002940.s002.pdf]

Phase I Study of the Safety and Immunogenicity of AMA1-C1/Alhydrogel® + CPG 7909, an Asexual Blood Stage Vaccine for Plasmodium falciparum Malaria.

DMID Protocol #: 04-075

FDA IND #: 12,029
Version 6.0, July 12, 2007

Sponsor: Division of Microbiology and Infectious Diseases (DMID)
National Institute of Allergy and Infectious Diseases (NIAID)
6610 Rockledge Drive, Bethesda, MD 20817

DMID Medical Monitor Shy Shorer, MD, RAC, CCRA
NIAID/DMID/OCRA
6610 Rockledge Dr
Bethesda, MD 20892
Telephone: 301-451-3025

DMID Protocol Champion Abdi Naficy, MD,
NIAID/DMID/PIPB
6610 Rockledge Dr
Bethesda, MD 20892
Telephone: 301-435-2866

Investigators: John J. Treanor, M.D. (Principal Investigator)
M. Christine Hay, M.D.
David Topham PhD
University of Rochester School of Medicine
601 Elmwood Avenue
Rochester, NY
Telephone: 585-275-5871

On site medical monitor: Peter Mariuz M.D.
Associate Professor of Medicine
University of Rochester School of Medicine
601 Elmwood Avenue
Rochester, NY
Telephone: 585-275-5871

Clinical Laboratory: Strong Memorial Hospital Clinical Laboratories
University of Rochester Medical Center
601 Elmwood Avenue
Rochester, NY
Telephone: 585-275-5871

Immunology Laboratories Malaria Vaccine Development Branch (MVDB)
National Institutes of Allergy and Infectious Diseases
5640 Fishers Lane, Twinbrook I
Rockville, MD 20852

Telephone: 301-435-3405

David Smith Center for Vaccine Biology
University of Rochester School of Medicine
Rochester, NY

SIGNATURE PAGE

The signature below constitutes the approval of this protocol and the attachments, and provides the necessary assurances that this trial will be conducted according to all stipulations of the protocol, including all statements regarding confidentiality, and according to local legal and regulatory requirements and applicable U.S. federal regulations and ICH guidelines.

The Lead PI, the PIs from all participating clinical sites and other key parties should sign the signature page as appropriate.

Principal Investigator:

Signed: ______________________________ Date: ____________

Name

Title

1 BACKGROUND INFORMATION 7
1.1 MALARIA VACCINES 7
1.2 PRELIMINARY STUDIES 8
1.3 NAME AND DESCRIPTION OF INVESTIGATIONAL PRODUCTS 10
1.4 KNOWN AND POTENTIAL RISKS AND BENEFITS 11
1.5 DOSAGE AND ROUTE OF ADMINISTRATION 12
1.6 CONDUCT OF THE STUDY 13
1.7 DESCRIPTION OF THE STUDY POPULATION 13
2 OBJECTIVES 13
2.1 PRIMARY OBJECTIVES 13
2.2 SECONDARY OBJECTIVES 14
2.3 TERTIARY OBJECTIVES: 14
3 TRIAL DESIGN AND METHODS 14
3.1 TRIAL ENDPOINTS 14
3.2 STUDY DESIGN 15
3.3 MEASURES TO AVOID BIAS 16
3.4 TRIAL TREATMENTS 16
3.5 DURATION OF SUBJECT PARTICIPATION 18
3.6 DISCONTINUATION OF SUBJECTS/STOPPING RULES 18
3.7 ACCOUNTABILITY OF PRODUCTS 18
3.8 MAINTENANCE OF STUDY CODES 18
3.9 DATA TO BE CONSIDERED SOURCE DATA 19
4 SUBJECTS: 19
4.1 INCLUSION CRITERIA 19
4.2 EXCLUSION CRITERIA: 19
4.3 ASSIGNMENT TO GROUPS 21
4.4 SUBJECT WITHDRAWAL/DISCONTINUATION 21
5 TREATMENT OF SUBJECTS 23
5.1 SCREENING (UP TO 56 DAYS PRIOR TO VACCINATION) 23
5.2 ENROLLMENT: 23
5.3 RANDOMIZATION 23
5.4 IMMUNIZATION PROCEDURE: 24
5.5 STUDY DAY 0 (DAY OF FIRST VACCINATION) 24
5.6 STUDY DAY 3 25
5.7 STUDY DAY 7 +/- 1 25
5.8 STUDY DAY 14 +/- 2 25
5.9 STUDY DAY 28 +/- 4 (DAY OF SECOND VACCINATION) 25
5.10 STUDY DAY 31 (3 DAYS AFTER SECOND VACCINATION) 26
5.11 STUDY DAY 35 +/- 1 (7 DAYS AFTER SECOND VACCINATION) 26
5.12 STUDY DAY 42 +/- 2 (14 DAYS AFTER SECOND VACCINATION) 26
5.13 STUDY DAY 56 +/- 4 (DAY OF THIRD VACCINATION) 26
5.14 STUDY DAY 59 (3 DAYS AFTER THIRD VACCINATION) 27
5.15 STUDY DAY 63 +/- 1 (7 DAYS AFTER THIRD VACCINATION) 27
5.16 STUDY DAY 70 +/- 2 (14 DAYS AFTER THIRD VACCINATION) 27
5.17 STUDY DAY 84 +/- 14 (28 DAYS AFTER THIRD VACCINATION) 28
5.18 STUDY DAY 140 +/- 14 (84 DAYS AFTER THIRD VACCINATION) 28
5.19 STUDY DAY 236+/- 30 (180 DAYS AFTER THIRD VACCINATION) 28
5.20 PLASMAPHERESIS 28
5.21 MONITORING SUBJECT COMPLIANCEPL 29
5.22 STUDY EVENTS FLOW SHEET 29
6 ASSESSMENT OF OUTCOME/IMMUNE RESPONSE 30
6.1 OUTCOME PARAMETERS 30
6.2 METHODS AND TIMING OF OUTCOME ASSESSMENTS 30
6.3 USE, STORAGE, AND TRACKING OF SPECIMENS AND DATA 32
6.4 RETENTION OF SPECIMENS FOR FUTURE USE 32

7 ASSESSMENT OF THE SAFETY OF VACCINATION....33
7.1 SAFETY PARAMETERS....33
7.2 METHODS AND TIMING OF SAFETY ASSESSMENTS....33
7.3 ADVERSE EVENTS, REACTOGENICITY, SERIOUS ADVERSE EVENTS....34
7.4 REPORTING PROCEDURES....36
7.5 PROCEDURES TO BE FOLLOWED IN THE EVENT OF ABNORMAL LABORATORY TEST VALUES OR ABNORMAL CLINICAL FINDINGS....38
7.6 TYPE AND DURATION OF THE FOLLOW-UP OF SUBJECTS AFTER ADVERSE EVENTS....38
7.7 HALTING RULES....38
8 CLINICAL MONITORING STRUCTURE....39
8.1 SITE MONITORING PLAN....39
9 BIOSTATISTICAL DESIGN AND ANALYSIS....40
9.1 STATISTICAL METHODS....40
9.2 ENDPOINTS....40
9.3 ANALYSIS PLAN....40
9.4 SAMPLE SIZE....42
9.5 MISSING DATA....42
9.6 DEVIATIONS FROM THE STATISTICAL PLAN....42
9.7 SELECTION OF SUBJECTS FOR ANALYSIS....42
10 DIRECT ACCESS TO SOURCE DATA/DOCUMENTS....42
11 QUALITY CONTROL AND QUALITY ASSURANCE....43
12 ETHICS/PROTECTION OF HUMAN SUBJECTS....43
12.1 DECLARATION OF HELSINKI....43
12.2 INSTITUTIONAL REVIEW BOARD....43
12.3 INFORMED CONSENT PROCESS....43
12.4 EXCLUSION OF WOMEN, MINORITIES, AND CHILDREN (SPECIAL POPULATIONS)....44
12.5 SUBJECT CONFIDENTIALITY....45
13 A HANDLING AND RECORDKEEPING....45
13.1 SOURCE DOCUMENTS AND ELECTRONIC CASE REPORT FORMS:....45
13.2 DATA MANAGEMENT RESPONSIBILITIES....45
13.3 DATA CAPTURE METHODS....46
13.4 TYPES OF DATA....46
13.5 TIMING/REPORTS....46
13.6 STUDY RECORDS RETENTION....46
13.7 PROTOCOL DEVIATIONS....47
14 FINANCING AND INSURANCE....47
15 PUBLICATION POLICY....47
16 LITERATURE CITED....48
17 APPENDICES....49
17.1 SUMMARY OF PRECLINICAL ANIMAL STUDIES....49
17.2 TOXICITY TABLES FOR SOLICITED ADVERSE EVENT INTENSITY....50
17.3 TOXICITY GRADING SCALE FOR LABORATORY ADVERSE EVENTS....51
17.5 COMMON TOXICITY CRITERIA FOR GRADING UNSOLICITED ADVERSE EVENTS....52

PROTOCOL SYNOPSIS

Purpose: This study will evaluate the safety and immunogenicity of the experimental malaria vaccine AMA1-C1/Alhydrogel®, and the ability of the TLR-9 agonist CPG 7909 oliogdexoynucleotide (ODN) to augment antibody responses to the vaccine and alter the Th1/Th2 bias. The vaccine preparations to be studied contain an equal mixture of AMA1 from two different clones of *Plasmodium falciparum* (FVO and 3D7), both produced separately as recombinant proteins expressed by *Pichia pastoris* (PpAMA-1 FVO and PpAMA-1 3D7). Bulk PpAMA-1 antigens were purified from culture medium of transformed yeast grown in a 60L fermenter. The correctly folded PpAMA-1 was purified from this mixture by a combination of affinity, ionic, hydrophobic and gel filtration chromatography. Purified PpAMA-1 FVO and PpAMA-1 3D7 were subsequently mixed and adsorbed onto aluminum hydroxide gel (Alhydrogel®). The CPG 7909 ODN formulation used in this study (CPG 7909) is manufactured by Coley Pharmaceutical Group. Subjects will be randomly assigned to receive Alhydrogel® formulated vaccine with or without CPG 7909 in a point of use formulation.

Research Environment: The study will be conducted at the University of Rochester Vaccine and Treatment Evaluation Unit (VTEU).

Subjects: Subjects for this study will be healthy adults between the ages of 18 and 45 years with no history of malaria or of recent travel to malaria-endemic areas. Subjects will be enrolled in three consecutive dose-escalation cohorts with review by a Safety Monitoring Committee between cohorts.

Subject Participation: Subjects will receive three vaccinations with the AMA1-C1/Alhydrogel® vaccine formulated in Alhydrogel® with or without CPG 7909 adjuvant over 2 months (0, 1, 2 months) by intramuscular (IM) injection. Subjects will have multiple blood samples obtained over the next 6 months.

Variables to be Investigated: Samples will be tested for binding antibody to AMA1 and for ability to inhibit the growth of plasmodia in vitro. In addition, antigen-specific activated B cells in peripheral blood will be enumerated, as well as the relative ratio of antigen-specific Th1-like and Th2-like T cells.

Risk/Benefits: The risks of participating in this study are those associated with administration of AMA1-C1/Alhydrogel® and of CPG 7909, and include local pain, systemic inflammatory responses including fever and influenza-like symptoms, and induction of autoimmune responses. As with any other investigational vaccine, there are unknown risks. Subjects may derive no benefit from participation in this study. Development of effective vaccines to prevent malaria is an important societal benefit.

Confidentiality: Volunteers will have code numbers and will not be identified by name.

1 BACKGROUND INFORMATION

1.1 MALARIA VACCINES

As reported by the World Health Organization in 2002, the worldwide incidence of malaria is approximately 300 million clinical cases annually, with approximately one million deaths per year attributed to malaria alone or in combination with other diseases [1]. Most of the mortality occurs among children under 5 years of age in sub Saharan Africa. Of the four species of malaria that infect humans, *Plasmodium falciparum* is responsible for the majority of these deaths. Mounting drug resistance of the malaria parasite, as well as widespread resistance of mosquitoes to insecticides make these control strategies increasingly unrealistic. A vaccine that would reduce both mortality and morbidity secondary to *P. falciparum* infection would be a valuable resource in the fight against this disease.

Apical membrane antigen-1 (AMA1) is produced by mature *P. falciparum* schizonts in infected erythrocytes [2], and localizes in the electron-dense neck of the rhoptry, an apical secretory organelle of the merozoite involved in red cell invasion [3]. The protein is subsequently exported to the merozoite surface at around the time of rupture of the schizont-infected erythrocyte [4]. AMA1 is not carried into the erythrocyte during invasion, but remains associated with the invasion interface and the areas of the merozoite that have not yet entered the erythrocyte [4, 5]. Although its exact function remains undetermined, these observations suggest that it performs a role during merozoite invasion of erythrocytes.

The AMA1-C1/Alhydrogel® vaccine has been tested in two human clinical trials under an U.S. F.D.A. Investigational New Drug Application (BB-IND-10944). The first Phase 1 trial in the U.S. demonstrated that the vaccine was well-tolerated. No safety concerns were identified. The immunogenicity data showed modest immune responses could be elicited in humans, and that functional antibody was present using the growth inhibition assay. The second Phase 1 trial started in Mali in May 2004. The adult subjects have received two vaccinations thus far, and a third booster vaccination will be administered 12 months after the first vaccination. No safety concerns related to vaccination in this study have been identified thus far.

Recently, it has been demonstrated that polynucleotides containing a CPG 7909 motif can significantly enhance B cell responses to co-administered antigens via interactions with toll-like receptor 9 (TLR-9). Therefore, this study will evaluate whether the addition of CPG 7909 will improve the antibody response to the AMA1-C1/Alhydrogel® vaccine. Additionally, CPG 7909 is known to induce a Th1-biased immune response which has been shown to be more

effective for inducing cell-mediated immune responses. CPG 7909 has been administered to humans on its own as an investigational cancer therapeutic agent and as a vaccine adjuvant with two licensed vaccines to healthy adult subjects without significant safety concerns under U.S. F.D.A. IND (BB-IND-11795).

1.2 PRELIMINARY STUDIES

1.2.1 Preclinical studies with AMA1-C1/Alhydrogel®

A total of 3 individual preclinical trials have been conducted to assess the safety and immunogenicity of the AMA1-C1/Alhydrogel® + CPG 7909 vaccine (see Appendix 17.1 for a tabular summary of the preclinical trials) prior to human clinical trials.

This combination has been tested by IM administration in mice, rats, and guinea pigs. The AMA1-C1/Alhydrogel® + CPG 7909 formulation was more immunogenic in the animals as compared to AMA1-C1/Alhydrogel® given without CPG 7909. No clinically significant adverse effects have been observed.

1.2.2 Clinical trials with AMA1-C1/Alhydrogel®

There have been two human clinical trials to date using the recombinant AMA1-C1/Alhydrogel® vaccine.

The first clinical trial to use AMA1-C1/Alhydrogel® was performed at Johns Hopkins University (Baltimore, MD) and is currently in volunteer follow up stages. The vaccine candidate which was tested at Hopkins is the same product to be used in this protocol without the CPG 7909. Thirty subjects have received up to 3 immunizations (Day 0, 28 and 180) with either 5, 20, or 80 µg doses of AMA1-C1/Alhydrogel® in an open label dose escalating study. No safety concerns were identified. Adverse events related to vaccination have been limited to local injection site reactions, which have been mild to moderate in severity. There was a dose response with the 80 µg group giving the highest antibody levels at day 42 (14 days after the second vaccination) and at day 194 (14 days after the third vaccination).

The second clinical trial using AMA1-C1/Alhydrogel® was started in Mali in May 2004. The study is a randomized double blind controlled trial. Fifty-four healthy Malian adults were randomized 2:1 to receive either AMA1-C1/Alhydrogel® at either 5, 20, or 80 µg doses, or a comparator vaccine. Thus far, each of the 3 dose groups have received two vaccinations. No safety concerns have been identified at this time.

Additionally, a recombinant AMA1 3D7 vaccine using Montanide ISA720 as the adjuvant has been tested in Australia. A Phase 1 study was conducted using the recombinant AMA1

ectodomain expressed in and refolded from *Escherichia coli*. Thirty-five healthy adults received either 5 µg (n=10), 20 µg (n=10), or 80 µg (n=9) of protein emulsified in Montanide ISA 720, or Montanide ISA 720 alone (n=6) as a control. Immunizations were administered as 0.5 mL IM injections; the second dose was given 2-3 months following the first dose, and the third dose was given 6 months following the second. The vaccine was generally well tolerated, although a number of subjects developed local AEs, in both the vaccine and control groups. A total of 32 AEs occurred that were possibly or probably related to the vaccine: one was systemic (headache and flushing after the first vaccination), and the remaining events were local reactions. The local AEs consisted mainly of pain and swelling at the injection site (13 mild, 17 moderate, and 1 severe). The formulation, a water-in-oil emulsion of antigen in Montanide ISA720, is not used in any approved vaccines, and is expected to elicit a significant proportion of local adverse reactions.

1.2.3 Previous clinical experience with aluminum-based adjuvants

Several licensed vaccines contain aluminum-based adjuvants, including the recombinant Hepatitis B vaccine (Recombivax HB®) and the diphtheria-tetanus toxoids vaccine (DT). Recombivax HB® may be a particularly useful comparator vaccine, as it consists of a recombinant protein expressed in *Saccharomyces cerevisiae* and is administered IM. For these two aluminum-adsorbed vaccines, local reactions such as pain, tenderness, and swelling are experienced in between 7.6% and 16.7% of subjects in studies that included over 1,200 healthy adults. Fever is seen in 3.2% to 9.3%, headache in 4.1%, and other systemic symptoms such as fatigue, malaise, nausea, and diarrhea at lower frequencies [6]. Urticaria has been reported in 0.1% of individuals vaccinated with Recombivax HB®. These are data based on the Recombivax HB® vaccine that also contained the preservative thimerosal, which may increase reactogenicity.

1.2.4 Previous clinical experience with CPG 7909 (VaxImmune®):

This CPG 7909 motif has been administered to humans in combination with the Engerix-B® Hepatitis B vaccine (in press) and the Fluarix® killed influenza vaccine (in press). Initial results from these two Phase 1 clinical trials indicate that the addition of CPG 7909 to these two licensed vaccines was safe, and in the case of Engerix-B®, induces significantly earlier and stronger antibody responses than the vaccine alone (Heather Davis, personal communication).

The Engerix-B® trial was a randomized, double-blind study in 56 healthy subjects in Canada. This was a dose escalating study comparing 0.125, 0.5, and 1.0 mg of CPG 7909 admixed with the licensed Engerix-B® vaccine. The most frequently reported adverse events

were injection site reactions (pain and erythema), flu-like symptoms and headache. All adverse events were mild or moderate in severity. One volunteer experienced a hypersensitivity-type reaction immediately following the third dose of Engerix-B® + 1.0 mg of CPG 7909. The symptoms included warmth, weakness, nausea, and dizziness. The symptoms resolved without treatment.

The Fluarix® trial was a randomized, controlled, double-blind study in 60 healthy subjects in Canada. Subjects received either the licensed Fluarix® vaccine with or without CPG 7909, or 1/10th the dose of Fluarix® with or without CPG 7909. The most frequently reported adverse events were injection site pain, headache, myalgia and fatigue. All injection site reactions were mild or moderate in severity, with the exception of one volunteer in the 1/10th dose Fluarix® + CPG 7909 group who reported severe pain which resolved without treatment within four days. Transient reductions in total WBC, neutrophils, lymphocytes, eosinophils and platelets were observed in all four study arms 2 days post-vaccination. None of these results were felt to be clinically significant.

One volunteer who received Engerix-B® + 1.0 mg of CPG 7909 had periodic elevations in anti-dsDNA which was initially detected two weeks after the second and third vaccinations. The anti-dsDNA returned to normal prior to receipt of the third dose and was normal at the end of the study. The volunteer was asymptomatic, and ANA and rheumatoid factor remained negative throughout.

1.3 NAME AND DESCRIPTION OF INVESTIGATIONAL PRODUCTS

1.3.1 AMA1-C1/Alhydrogel® Malaria Vaccine.

Both recombinant AMA1 FVO and AMA1 3D7 are highly purified 62 kDa proteins that correspond to the ectodomain of *P. falciparum* (FVO) AMA1 and *P. falciparum* (3D7) AMA1, respectively. AMA1 FVO and AMA1 3D7 each consist of the ectodomain of the mature protein found in parasites with the addition of a 6-histidine C-terminal tag to allow purification of the protein. AMA1 FVO and AMA1 3D7 were purified from the fermentation supernatant using a combination of affinity, ionic, hydrophobic, and gel filtration chromatography. The purification process was designed to separate full-length product from degraded material as well as non-product-related contaminants. AMA1 FVO and AMA1 3D7 bulk antigens were both manufactured at the WRAIR Bioproduction Facility (Silver Spring, Maryland) according to cGMP. The bulk antigens were prepared in unbuffered, isotonic saline without preservatives.

1.3.2 Alhydrogel®

Aluminum hydroxide gel (HCI Biosector, Denmark) has been extensively used as an adjuvant in many licensed human vaccines. Aluminum-containing adjuvants are in routine human use and contained in many licensed human vaccines. Alhydrogel® is supplied sterile, in isotonic saline without preservatives.

1.3.3 CPG 7909 Oligodeoxynucleotide

CPG 7909 (Coley Pharmaceutical Group, Wellesley, MA) is an investigational agent under U.S. F.D.A I.N.D (BB-IND-11795). CPG 7909 is a short synthetic oligodeoxynucleotide of the following sequence: 5'-TCG TCG TTT TGT CGT TTT TTT CGA-3', with all nucleotides linked with phosphorothioate bonds. CPG 7909 (VaxImmune) is manufactured according to cGMP standards and is supplied in sterile vials at 10 mg/mL in phosphate buffered saline for IM administration.

1.4 KNOWN AND POTENTIAL RISKS AND BENEFITS

Possible local vaccine reactions include pain, swelling, erythema, induration, limitation of limb movement for several days, lymphadenopathy, or pruritus at the injection site. Local SC nodules, believed to be granulomatous reactions to aluminum hydroxide, have been observed with use of aluminum hydroxide-based adjuvants. Thus, most aluminum hydroxide-adsorbed vaccines are injected IM rather than SC. Systemic reactions such as fever, chills, headache, fatigue, malaise, myalgia, joint pain, may also possibly occur. Immediate hypersensitivity reactions including urticaria, anaphylaxis, or other IgE mediated responses are possible as with any vaccine.

CPG 7909 is a potent immune activator, and has the potential to overcome the normal tolerance of the immune system for self antigens. In a number of murine models, administration of CPG 7909 has been associated with development of certain autoimmune phenomenon (see Coley Investigator Brochure, Appendix 3). However, the relevance of this finding to humans is unclear because while TLR-9 receptors are widely distributed among immune cells in mice, they appear to be restricted to B cells and plasmacytoid dendritic cells in humans. No subject in any study to date with CPG 7909 oligonucleotides has developed signs or symptoms of autoimmune disease.

In animal studies, CPG 7909 oligodeoxynucleotides (ODN) given by themselves only rarely induce the production of anti-dsDNA antibodies. However, if the CPG 7909 ODN are administered as a complex together with a foreign protein or other antigen that can elicit T cell help, then it is likely that antibodies against the CPG 7909 ODN will be produced, and these

may cross-react with dsDNA. Vaccination of humans with a CPG 7909 ODN together with an antigen capable of binding to the ODN could be expected to result in the production of IgG anti-ODN antibodies, which could cross react with self DNA.

This phenomenon may have been observed in clinical studies with CPG 7909. One normal subject in the Hepatitis B study, and 27/155 subjects in studies involving use of CPG 7909 as an antineoplastic agent, have developed detectable antibody to double-stranded DNA. The specificity of these antibodies for ss or ds DNA has not been determined at this time, and the reactivity of the antibody with the CPG 7909 ODN itself has not been determined. These antibodies have generally declined to baseline after cessation of CPG 7909, and have not been associated with other autoimmune phenomenon, and their possible clinical significance is unknown. Subjects in this study will be closely monitored for laboratory evidence or clinical signs or symptoms of autoimmunity.

As with any investigational vaccine, there is a theoretical possibility of risks about which we have no present knowledge. Subjects will be informed of any such risks should further data become available.

Female participants will be cautioned of the unknown risk of study vaccines to the fetus and will be advised to use adequate birth control methods for the duration of the study.

Risks occasionally associated with venipuncture include pain and bruising at the site of venipuncture, lightheadedness, and syncope (rarely).

Subjects will not receive any direct benefit from participation in this study. It is hoped that information gained in this study will contribute to the development of a safe and effective malaria vaccine.

1.5 DOSAGE AND ROUTE OF ADMINISTRATION

Subjects in this study will be randomized to receive either

Group A: 20 µg AMA1-C1/Alhydrogel® with 377 µg Aluminum and 500 µg CPG 7909

Group B: 80 µg AMA1-C1/Alhydrogel® with 368 µg Aluminum and 500 µg CPG 7909

Group C: 80 µg AMA1-C1/Alhydrogel® with 368 µg Aluminum alone

All groups will receive three injections separated by one month between injections of the same dose level. All doses will be given by IM injection in the deltoid muscle in alternating arms.

1.6 CONDUCT OF THE STUDY

The investigator(s) must adhere to the protocol as detailed in this document. The Principal investigator will be responsible for enrolling only those volunteers who have met protocol eligibility criteria.

A physician may implement a deviation from, or a change in, the protocol to eliminate immediate hazard(s) to trial subjects without prior NIAID and/or IRB/IEC approval. As soon as possible, the implemented deviation or change, the reasons for it and if appropriate, the proposed protocol amendment(s) must be submitted:

- To the IRB/IEC for review and approval/favorable decision
- To NIAID for agreement; and if required
- To the appropriate regulatory authorities.

Any physician-implemented changes as described above and all other protocol violations must be reported to the Data Coordinating Center in a timely manner. Protocol violations that result in an adverse event must be reported on an AE/SAE form and on the Protocol Deviation form and faxed to PPD at 1-888-488-9697 within 72 hours of site awareness. The Data Coordinating Center must be notified by submission of the form in the Internet Data Entry System (IDES) within 72 hours for all deviations. The following items must be addressed on the Protocol Violation/Deviation CRF or contained in supplemental documentation attached to the CRF, as appropriate:

- Nature of the error
- Standard reporting information (subject's clinical status before and after the event)
- Steps taken to review the error
- Steps taken to assure the error will not occur again.

Protocol violations must also be reported to the site's IRB in accordance with IRB policies.

1.7 DESCRIPTION OF THE STUDY POPULATION

The study will be conducted in healthy adults ages 18 to 45 with no history of malaria or recent travel to malarious areas.

2 OBJECTIVES

2.1 PRIMARY OBJECTIVES

2.1.1 To assess the safety, reactogenicity of the AMA1-C1/Alhydrogel® + CPG 7909 vaccine.

2.2 SECONDARY OBJECTIVES

2.2.1 To demonstrate that the addition of CPG 7909 improves the immune responses to AMA1-FVO and AMA1-3D7, as compared to AMA1-C1/Alhydrogel® at day 70 (14 days after 3rd vaccination).

2.2.2 To determine the dose of AMA1-C1/Alhydrogel® + CPG 7909 that generates the highest serum antibody levels of AMA1-FVO and AMA1-3D7 at day 70.

2.2.3 To assess and compare the duration of antibody responses to AMA1-FVO and AMA1-3D7 proteins by enzyme-linked immunosorbent assay (ELISA) over an 8 month period (6 months after 3rd vaccination).

2.2.4 To perform exploratory studies of B and T cell populations both before and after vaccination.

2.3 TERTIARY OBJECTIVES:

The following objectives are for informational purposes only:

2.3.1 To measure the biological activity of the antisera by an in vitro parasite growth inhibition assay using FVO and 3D7 parasites.

2.3.2 To determine the relationship between anti-AMA1 antibody concentration as determined by ELISA and the degree of in vitro growth inhibition of *P. falciparum*.

2.3.3 To determine the relative specificity of the antibodies to a range of AMA1 serotypes in addition to FVO and 3D7, as judged by ELISA and growth inhibition on a select panel of parasites with typed AMA1 antigens.

3 TRIAL DESIGN AND METHODS

3.1 TRIAL ENDPOINTS

The trial endpoints are described below:

3.1.1 Safety

Safety endpoints include the frequency and severity of local and systemic adverse events after each dose reported on the subject diary card (solicited adverse events) or reported by subjects spontaneously (unsolicited adverse events) and the presence of abnormal clinical laboratory tests after immunization in each group.

3.1.2 Immunogenicity

Immunogenicity endpoints include the frequency and titer of vaccine induced neutralizing and binding antibody, and the presence and quantity of antigen-specific B cells and CD4 and CD8 T cells producing specified cytokines following vaccination.

3.2 STUDY DESIGN

The study is a dose escalating Phase 1 clinical trial in healthy adult subjects designed to evaluate the safety, reactogenicity, and immunogenicity of the AMA1-C1 malaria vaccine formulated on Alhydrogel® co-administered with CPG 7909. The subjects will be randomized within group to receive one of 2 doses of AMA1-C1/Alhydrogel® + CPG 7909 or 80 µg of AMA1-C1/Alhydrogel® without CPG 7909. The subjects will be blinded to which vaccine they are receiving, AMA1-C1/Alhydrogel® with or without CPG 7909. This study will be staggered such that dose escalation will not occur until the lower dose group has received two vaccinations at days 0 and 28. The safety data up to and including day 35 (7 days post-vaccination #2) will be reviewed prior to dose escalation by the Safety Monitoring Committee.

After providing written informed consent, subjects will undergo eligibility screening, including medical history, physical examination, hematology testing, liver and renal function testing, HIV, Hepatitis B and C screening, rheumatoid factor, anti-dsDNA and ANA testing, and urinalysis; pregnancy testing will be performed on female subjects. For participants who are eligible, the Day 0 visit will be scheduled to receive the first dose of vaccine. Subjects will be observed for immediate reactions following each vaccination for 30 minutes. Subjects will return to the clinic on Days 3, 7 and 14 following each vaccination for clinical assessment.

Seventy-five (75) subjects will be progressively enrolled into one of 3 cohorts. The first 20 subjects (Cohort 1) will be randomized at a 3:1 ratio to receive either the 20 µg dose of AMA1-C1/Alhydrogel® + CPG 7909 or the 80 µg AMA1-C1/Alhydrogel® vaccine. After review of safety data at day 35 by the Safety Monitoring Committee (SMC) the study will proceed to Cohort 2. In Cohort 2, the next 20 subjects will be randomized 3:1 to receive either the 80 µg

dose of AMA1-C1/Alhydrogel® + CPG 7909 or the 80 µg AMA1-C1/Alhydrogel® vaccine, as outlined in the table below.

		Immunization Schedule		
Cohort	Total Volunteers	Day 0	Day 28	Day 56
1	20	A (15) or C (5)	A (15) or C (5)	A (15) or C (5)
2	20	B (15) or C (5)	B (15) or C (5)	B (15) or C (5)
3	35	B (15) or C (20)	B (15) or C (20)	B (15) or C (20)
TOTAL	75	A: 20 µg AMA1-C1/424 µg Alhydrogel® + 500 µg CPG 7909<br>B: 80 µg AMA1-C1/424 µg Alhydrogel® + 500 µg CPG 7909<br>C: 80 µg AMA1-C1/424 µg Alhydrogel®		

After evaluation of 35-day safety data in Cohort 2, and evaluation of all available safety data in Cohort 1, an additional 35 subjects (Cohort 3) will be randomized to receive the 80 µg AMA1-C1/Alhydrogel® either with or without CPG 7909 in a 3:4 ratio.

A Safety Monitoring Committee (SMC) will be asked to review cumulative safety data prior to dose escalation. Stopping rules for dose escalation are given in section 7.7.

A total of seventy five subjects will be enrolled. Forty five subjects will receive the AMA1-C1/Alhydrogel® + CPG 7909 vaccine (one of two dose concentrations), and 30 will receive the 80 µg AMA1-C1/Alhydrogel® vaccine.

3.3 MEASURES TO AVOID BIAS

Subjects within each cohort will be blinded as to study assignment.

3.4 TRIAL TREATMENTS

AMA1-C1/Alhydrogel® vaccine will be supplied to the study-site pharmacist by the Pharmaceutical Development Section, Pharmacy Department, Clinical Center, National Institutes of Health, where the vaccine was formulated and vialed. Temperatures during vaccine transport will remain between 0.5°C and 9°C. The site pharmacist will label the kit with the assigned Volunteer ID number and randomization code. The vaccine should be stored in the refrigerator at 2°C to 8°C and should not be frozen. Single-dose vials should be stored in the upright position. CPG 7909 will be supplied to the study-site pharmacist by the Malaria

Vaccine Development Branch, who obtained the adjuvant through a clinical trials agreement with Coley Pharmaceutical Group, Inc.

3.4.1 AMA1-C1/Alhydrogel®

3.4.1.1 Formulation and Dosage

AMA1-C1/Alhydrogel® malaria vaccine is supplied as a cloudy suspension in single dose vials. Each 2.0 mL vial contains 0.7 mL, of which 0.5 mL is the intended volume to be injected. 0.5 mL of vaccine contains up to the equivalent of 377 µg of aluminum as Alhydrogel® (800 µg of aluminum hydroxide gel per dose) onto which either 20 µg, or 80 µg of recombinant AMA1-C1 has been bound. The product conforms to established requirements for sterility, safety, and identity

3.4.1.2 Packaging and Labeling

AMA1-C1/Alhydrogel® malaria vaccine will be packaged in unit dose vials labeled for investigational use only.

3.4.1.3 Supply, Storage, Handling and Disposal

AMA1-C1/Alhydrogel® vaccine should be maintained at 2°C to 8°C until just prior to administration. Vaccine should NOT be frozen at any time.

After administration of a vaccine dose, the single-dose vial will be returned to the Pharmacy at the test site, and vials will be accounted for and stored until monitoring by the IND sponsor. The vials may then be disposed of according to site protocol. At the conclusion of vaccine administration, all unused vaccine supplies will be returned to the MVDB.

3.4.1.4 Administration

AMA1-C1/Alhydrogel® malaria vaccine is administered in the deltoid muscle by IM injection.

3.4.2 CPG 7909

3.4.2.1 Formulation and Dosage

The trial site pharmacy will prepare the point of injection formulation as follows. CPG 7909 is supplied as a 1.0 mL solution in 2.0 mL multi-dose vials containing 10 mg/mL without preservative. A volume of 0.08 mL of CPG 7909 (10 mg/mL) will be withdrawn from the multi-dose vial of CPG 7909 using a 300 µL insulin syringe. The 0.08 mL of CPG 7909 will be added to a vial containing 0.7 mL of AMA1-C1/Alhydrogel®. This point of injection formulation should yield a final injection volume of 0.55 mL to be withdrawn for each vaccination. The actual dose of injected CPG 7909 will therefore be 0.56 mg. The mixture must be administered not more than 6 hours after mixing the CPG 7909 with the AMA1-C1/Alhydrogel®.

3.4.2.2 Packaging and Labeling

CPG 7909 will be supplied in multi-dose vials labeled for investigational use only.

3.4.2.3 Supply, Storage, Handling, and Disposal

CPG 7909 should be maintained at 2°C to 8°C until just prior to administration. CPG 7909 should NOT be frozen at any time.

3.4.2.4 Administration

After admixture with AMA1-C1/Alhydrogel®, administration will be by IM injection in to the deltoid muscle.

3.4.3 Point of administration formulation of AMA1-C1/Alhydrogel® + CPG 7909

Using a 300 µL insulin syringe, 0.08 mL of CPG 7909 (10 mg/mL) will be added to a 0.7 mL vial of AMA1-C1/Alhydrogel® as a point of injection formulation to yield a final injection volume of 0.55 mL for each vaccination. The actual dose of injected CPG 7909 will therefore be 0.56 mg. The mixture must be administered not more than 6 hours after formulation, and should remain at 2°C to 8°C until just prior to administration.

3.5 DURATION OF SUBJECT PARTICIPATION

Subjects will be followed routinely for a maximum of 180 (± 5) days following the third vaccination. Since subjects must be screened within 56 days of vaccination, the maximum duration of subject participation in the absence of continuing adverse events is 236 (± 5) days. Subjects with adverse events will be followed to resolution or stabilization if resolution is not expected.

3.6 DISCONTINUATION OF SUBJECTS / STOPPING RULES

Circumstances under which subjects may be discontinued from the study are described in section 4.4.

3.7 ACCOUNTABILITY OF PRODUCTS

After receipt of the vaccine and/or CPG 7909, the principal investigator is responsible for distribution and has ultimate responsibility for drug accountability. A log of receipt, temperature maintenance and disposal of vaccine and CPG 7909 will be maintained by the study pharmacist.

3.8 MAINTENANCE OF STUDY CODES

Study codes will be maintained by the study pharmacist. A backup, sealed copy of the study code will be maintained by the on-site medical monitor.

3.9 DATA TO BE CONSIDERED SOURCE DATA

Study data will be entered directly onto hard copies of the eCRF, which together with paper copies of laboratory reports, will be considered the source data for the trial.

4 SUBJECTS:

4.1 INCLUSION CRITERIA

4.1.1 Age between 18 and 45 years, inclusive.

4.1.2 Good general health as a result of review of medical history and/or clinical tests.

4.1.3 Available for the duration of the trial (34 weeks).

4.1.4 Willingness to participate in the study as evidenced by signing the informed consent document.

4.2 EXCLUSION CRITERIA:

4.2.1 Pregnancy as determined by a positive urine β-hCG (if female) at any point during the study.

4.2.2 Participant unwilling to use highly effective contraception methods (such as: abstinence, birth control pills or birth control patches or vaginal ring, diaphragm with spermicide, IUD (intrauterine device), condom with spermicide, progestin implant or injection, surgical sterilization (hysterectomy, bilateral oophorectomy, tubal ligation), or a partner who has had a vasectomy) for the duration of the trial (if female).

4.2.3 Currently lactating and breast-feeding (if female).

4.2.4 Evidence of clinically significant immunosuppressive, neurologic, cardiac, pulmonary, hepatic, rheumatologic, autoimmune, or renal disease by history,

physical examination, and/or laboratory studies including urinalysis (see Appendix 17.3).

4.2.5 Behavioral, cognitive, or psychiatric disease that in the opinion of the investigator affects the ability of the volunteer to understand and cooperate with the study protocol.

4.2.6 Laboratory evidence of liver disease (aspartate aminotransferase greater than 1.25 times the upper limit of normal of the testing laboratory).

4.2.7 Laboratory evidence of renal disease (serum creatinine greater than the upper limit of normal of the testing laboratory).

4.2.8 Laboratory evidence of hematologic disease (absolute neutrophil count <1,500/mm3; hemoglobin < 0.9 times the lower limit of normal of the testing laboratory, by sex; or platelet count <140,000/mm3).

4.2.9 Other condition that in the opinion of the investigator would jeopardize the safety or rights of a volunteer participating in the trial or would render the subject unable to comply with the protocol.

4.2.10 Participation in another investigational vaccine or drug trial within 30 days of starting this study, or while this study is ongoing.

4.2.11 Volunteer has had medical, occupational, or family problems as a result of alcohol or illicit drug use during the past 12 months.

4.2.12 History of a severe allergic reaction or anaphylaxis.

4.2.13 Severe asthma (emergency room visit or hospitalization within the last 6 months).

4.2.14 Serologic evidence of infection with HIV-1, HBV, or HCV.

4.2.15 Use of corticosteroids (excluding topical or nasal) or immunosuppressive drugs within 30 days of starting this study or while the study is ongoing.

4.2.16 Receipt of a live vaccine within past 4 weeks or a killed vaccine within past 2 weeks prior to entry into the study.

4.2.17 History of a surgical splenectomy.

4.2.18 Receipt of blood products within the past 6 months.

4.2.19 Previous receipt of an investigational malaria vaccine.

4.2.20 Receipt of antimalarial prophylaxis during the past 12 months.

4.2.21 Prior malaria infection.

4.2.22 Travel to a malaria-endemic country during the past 12 months or planned travel to a malaria-endemic country during the course of the study.

4.2.23 History of a known allergy to nickel.

4.2.24 Pre-existing autoimmune or antibody mediated diseases including but not limited to: systemic lupus erythematosis, rheumatoid arthritis, multiple sclerosis, Sjogren's syndrome, autoimmune thrombocytopenia; or laboratory evidence of possible autoimmune disease determined by a positive anti-dsDNA titer, positive rheumatoid factor, proteinuria and/or a positive ANA.

4.2.25 Chloroquine and related compounds within 12 weeks of study entry.

4.3 ASSIGNMENT TO GROUPS

The three stages will be enrolled consecutively, with movement from one stage to the next dependent on assessment of day 35 safety data from the previous stage. In stage 1, subjects will be randomized to receive either 20 µg of AMA1-C1/Alhydrogel® with CPG 7909 or 80 µg of AMA1-C1/Alhydrogel® alone, and in stage 2, subjects will be randomized to receive 80 µg AMA1-C1/Alhydrogel® with or without CPG 7909. In stage 3, additional subjects will be randomized to 80 µg AMA1-C1/Alhydrogel® with or without CPG 7909.

4.4 SUBJECT WITHDRAWAL/DISCONTINUATION

Individual subjects will not receive further doses of vaccine if they meet any of the following criteria:

4.4.1 Treatments that could potentially interfere with vaccine-induced immunity:

The following criteria should be checked at each visit. If any become applicable during the study, the participant will be excluded from receiving further doses of the study vaccine and will not be included in the immunogenicity evaluations. The participant will, however, be encouraged to remain in the safety evaluation for doses already received.

1. Use of any investigational drug or vaccine other than the study vaccine during the study period.
2. Administration of chronic (defined as more than 14 days) immunosuppressants or other immune-modifying drugs within six months of vaccination. (Topical steroids are allowed.)
3. Administration of immunoglobulins and/or any blood products up to 30 days after the last dose of vaccine.

4.4.2 Development of Contraindications to vaccination:

The following criteria should be checked prior to each immunization and are contraindications to further immunization. However, the participant will be encouraged to remain in the safety evaluation for doses already received.

1. Hypersensitivity reaction following administration of the study vaccine.
2. Positive urine ß-hCG.
3. Development of a serious or severe, vaccine-associated adverse event.
4. Development of proteinuria or positive antibody to dsDNA in the setting of a newly positive ANA.

4.4.3 Indications for deferral of vaccination:

The following events constitute grounds for deferral of vaccine administration at that point in time; if any one of these adverse events occurs at the time scheduled for vaccination, the participant may be vaccinated at a later date, within the allowable time interval specified in the protocol, or withdrawn at the discretion of the investigator. The participant must be followed until resolution of the event as with any adverse event. If the participant is withdrawn from the study, he/she will be encouraged to remain in the safety evaluation for the duration of the study.

1. Oral temperature > 37.5 °C at the time of vaccination will warrant deferral of immunization until fever and symptoms resolve.
2. Any other condition that in the opinion of the investigator poses a threat to the individual if immunized or that may complicate interpretation of the safety of the vaccine following immunization.

Such individual(s) will be followed until the symptoms resolve or the window for immunization expires. No further vaccination will be performed if the participant does not recover (oral temperature $\leq$ 37.5°C and/or lack of symptoms) within 7 days of the originally scheduled vaccination date. The participant, however, will be followed for safety and immunogenicity. If the individual meets any of the above criteria for deferral on the day of first immunization the investigator may elect to exclude the participant from further participation in the study.

If a subject withdraws or is withdrawn prior to completion of the study, the reason for this decision must be recorded in the case report forms. If a subject is withdrawn because of an AE or SAE, the subject must be followed until resolution or stabilization of the event.

5 TREATMENT OF SUBJECTS

5.1 SCREENING (UP TO 56 DAYS PRIOR TO VACCINATION)

1. Explain the study and Informed Consent form to the volunteer.
2. Ensure the subject has signed the Informed Consent form and HIV testing informed consent form and receives a signed copy of both consent forms.
3. Elicit a complete medical history, including menstrual and contraceptive history and/or history of surgical sterility for female subjects.
4. Administer a complete physical examination.
5. Obtain blood for hematology, biochemistry, anti-dsDNA, ANA, rheumatoid factor, and serologic tests for viral hepatitis and HIV in all subjects.
6. Obtain urine for urine dipstick testing, as well as β-HCG testing in females.
7. Counsel females to avoid becoming pregnant during the study.

5.2 ENROLLMENT:

Subjects who meet all entry criteria will be invited to return to the study site to enroll in the study. At that time, all procedures will be reviewed with the subject, and if agreeable, the subject will be randomized and vaccinated.

5.3 RANDOMIZATION

The eligible subjects assigned to each of the three cohorts will be asked to come to the clinic for their scheduled day 0 visit. After a history and physical exam is performed to ensure

that they are eligible for participation in the study, blood will be collected for the studies outlined in Section 5.21. Study participants will be assigned a unique study number.

Randomization to either the AMA1-C1/Alhydrogel® + CPG 7909 vaccine or 80 µg AMA1-C1/Alhydrogel® vaccine will be done online using the enrollment module of the Internet Data Entry System (IDES). The randomization codes will be included in the enrollment module for the trial. Each subject enrolled into the trial is assigned a treatment code after demographic and eligibility data have been entered into the system. The site will be provided with a code list to be kept in a secure place.

5.4 IMMUNIZATION PROCEDURE:

Subjects will receive three immunizations, on Days 0, 28, and 56. 0.50 mL of AMA1-C1/Alhydrogel® or 0.55 mL of AMA1-C1/Alhdyrogel® + CPG 7909 will be delivered by IM in the deltoid muscle with a 22-gauge needle of appropriate length after preparation of the site with alcohol. Successive vaccinations will be given in alternating arms.

5.5 STUDY DAY 0 (DAY OF FIRST VACCINATION)

1. Verify that Informed Consent was obtained.
2. Verify that all applicable eligibility criteria have been met.
3. Perform abbreviated history and physical exam, focusing the history and physical exam on any acute complaints.
4. Obtain blood for hematology, biochemistry, rheumatoid factor, anti-dsDNA, ANA, C3, C4, CH50, anti-AMA-1 antibody ELISA, B and T cell analysis, and *P. falciparum* growth inhibition assay (GIA).
5. Obtain urine for urine dipstick testing.
6. For females, obtain a urine sample for ß-hCG testing. Ensure that test is negative before proceeding; a positive test will exclude the volunteer from the trial.
7. Record vital signs (blood pressure, temperature, heart rate and respiratory rate).
8. Administer the vaccine.
9. Observe for 30 minutes after vaccination to evaluate for immediate adverse reactions.
10. Give subject oral thermometer, diary card and instructions for completion.

5.6 STUDY DAY 3

1. Perform interim history and physical exam (abbreviated H&P to include: injection site, skin, lymph nodes, and other body systems felt to be necessary by examining clinician), emphasizing examination of any acute complaints.
2. Record vital signs.
3. Obtain blood for hematology and biochemistry tests, and B and T cell analysis.
4. Review diary card.

5.7 STUDY DAY 7 + / - 1

1. Perform interim history and examination of the injection site, and physical exam if indicated by acute complaints.
2. Record vital signs.
3. Obtain blood for hematology, rheumatoid factor, anti-dsDNA, ANA, and B and T cell analysis.
4. Collect Day 0-6 diary card.

5.8 STUDY DAY 14 + / - 2

1. Perform interim history and examination of the injection site, and physical exam if indicated by acute complaints.
2. Record vital signs.
3. Obtain blood for hematology, biochemistry, and anti-AMA-1 antibody ELISA.
4. Obtain urine for urine dipstick testing.

5.9 STUDY DAY 28 + / - 4 (DAY OF SECOND VACCINATION)

1. Perform interim history and physical exam, emphasizing examination of any acute complaints.
2. Obtain blood for hematology, biochemistry, rheumatoid factor, anti-dsDNA, ANA, anti-AMA-1 antibody ELISA, and B and T cell analysis.
3. Obtain urine for urine dipstick testing.
4. For females, obtain a urine sample for β-hCG testing. Ensure that test is negative before proceeding; a positive test will exclude the volunteer from receiving further vaccinations.
5. Record vital signs.
6. Administer the vaccine.

7. Observe for 30 minutes after vaccination to evaluate for immediate adverse reactions.
8. Give subject oral thermometer, diary card and instructions for completion

5.10 STUDY DAY 31 (3 DAYS AFTER SECOND VACCINATION)

1. Perform abbreviated history and physical exam (abbreviated H&P to include: injection site, skin, lymph nodes, and other body systems felt to be necessary by examining clinician), emphasizing examination of any acute complaints.
2. Record vital signs.
3. Obtain blood for hematology and biochemistry tests, and B and T cell analysis.
4. Review diary card,

5.11 STUDY DAY 35 + / - 1 (7 DAYS AFTER SECOND VACCINATION)

1. Perform interim history and examination of the injection site, and physical exam if indicated by acute complaints.
2. Record vital signs.
3. Obtain blood for hematology and B and T cell analysis, and rheumatoid factor, anti-dsDNA, and ANA.
4. Collect Day 0-6 diary card.

5.12 STUDY DAY 42 + / - 2 (14 DAYS AFTER SECOND VACCINATION)

1. Perform interim history and examination of the injection site, and physical exam if indicated by acute complaints.
2. Record vital signs.
3. Obtain blood for hematology, biochemistry, anti-AMA-1 antibody ELISA, and GIA.
4. Obtain urine for urine dipstick testing

5.13 STUDY DAY 56 + / - 4 (DAY OF THIRD VACCINATION)

1. Perform interim history and physical exam, emphasizing examination of any acute complaints.
2. Obtain blood for hematology, biochemistry, rheumatoid factor, anti-dsDNA, ANA, anti-AMA-1 antibody ELISA, GIA and B and T cell analysis.
3. Obtain urine for urine dipstick testing.

4. For females, obtain a urine sample for β-hCG testing. Ensure that test is negative before proceeding; a positive test will exclude the volunteer from receiving further vaccinations.
5. Record vital signs
6. Administer the vaccine.
7. Observe for 30 minutes after vaccination to evaluate for immediate adverse reactions.
8. Give subject oral thermometer, diary card and instructions for completion

5.14 Study Day 59 (3 days after Third Vaccination)

1. Perform abbreviated history and physical exam (abbreviated H&P to include: injection site, skin, lymph nodes, and other body systems felt to be necessary by examining clinician), emphasizing examination of any acute complaints.
2. Record vital signs.
3. Obtain blood for hematology and biochemistry tests, and B and T cell analysis.
4. Review diary card.

5.15 Study Day 63 + / - 1 (7 days after Third Vaccination)

1. Perform interim history and examination of the injection site, and physical exam if indicated by acute complaints.
2. Record vital signs.
3. Obtain approximately blood for hematology, rheumatoid factor, anti-dsDNA, ANA, and B and T cell analysis.
4. Collect Day 0-6 diary card.

5.16 Study Day 70 + / - 2 (14 days after Third Vaccination)

1. Perform interim history and examination of the injection site, and physical exam if indicated by acute complaints.
2. Record vital signs.
3. Obtain blood for hematology, biochemistry, anti-AMA-1 antibody ELISA, and GIA.
4. Obtain urine for urine dipstick testing.

5.17 STUDY DAY 84 + / - 14 (28 DAYS AFTER THIRD VACCINATION)

1. Perform interim history and examination of the injection site, and physical exam if indicated by acute complaints.
2. Obtain blood for hematology, rheumatoid factor, anti-dsDNA, ANA, B and T cell analysis, anti-AMA-1 antibody ELISA.
3. Obtain urine for urine dipstick testing.

5.18 STUDY DAY 140 + / - 14 (84 DAYS AFTER THIRD VACCINATION)

1. Perform interim history and perform physical exam if indicated by complaints or history.
2. Obtain blood for hematology, B and T cell analysis, anti-AMA-1 antibody ELISA.

5.19 STUDY DAY 236+ / - 30 (180 DAYS AFTER THIRD VACCINATION)

1. Perform interim history and perform physical exam.
2. Record vital signs.
3. Obtain blood for hematology, rheumatoid factor, anti-dsDNA, ANA, B and T cell analysis, anti-AMA-1 antibody ELISA and GIA.
4. Obtain urine for urine dipstick testing.

5.20 PLASMAPHERESIS

Plasmaphereis will be performed on subjects who develop high levels of AMA-1 antibody and who agree to this procedure. The purpose of plasmapheresis will be to collect serum to serve as an antibody standard for future trials. The criteria for selection as a potential plasmapheresis donor will be the development of a serum AMA-1 antibody level of greater than 2000 AMA-3D7 MVDB ELISA units on Day 42. These subjects will be asked if they would be willing to undergo plasmapheresis, and if agreeable, will sign a separate consent for this procedure. If possible, up to 5 subjects will participate. Participation in the plasmapheresis component of the study does not otherwise impact participation in the main study.

Plasmapheresis will be performed by standard techniques by qualified personnel. The procedure will entail the removal of blood, the separation of 250-500 mL of plasma, and the reinfusion of red blood cells with calcium citrate and electrolytes, as per standard plasmapheresis procedures. Because all red blood cells are re-infused, no blood volume is lost and the total amount of blood drawn during the study remains unchanged.

5.21 MONITORING SUBJECT COMPLIANCEPL

Compliance with the study protocol and protocol mandated study visits will be recorded in the case report form. Subjects who miss study visits will be called and urged to return but will not be dropped from the study once they have already been vaccinated.

5.22 STUDY EVENTS FLOW SHEET

The following table summarizes the events in the study, and estimates the total volumes of blood drawn at each visit and in total.

			V1	V2	V3	V4	V5	V6	V7	V8	V9	V10	V11	V12	V13	V14	V15
	BV*	Pre	0	3	7	14	28	31	35	42	56	59	63	70	84	140	236
Hx and Exam		X															
Informed consent		X															
Interim Hx, Exam			X	X	X	X	X	X	X	X	X	X	X	X	X	X	X
CBC & differential**	5	X	X	X	X	X	X	X	X	X	X	X	X	X	X	X	X
Chemistries	5	X	X	X		X	X	X		X	X	X		X			
Urinalysis		X	X			X	X			X	X			X	X		X
Urine pregnancy***		X	X				X				X						
HIV, HBV, HCV	10	X															
RF, Anti-dsDNA, ANA	5	X	X		X		X		X		X		X		X		X
C3, C4, CH50	5		X														
VACCINATION			X				X				X						
Anti-AMA1 antibody	10		X			X	X			X	X			X	X	X	X
GIA	20		X							X	X			X			X
B cell analysis	20		X	X	X		X	X	X		X	X	X	X	X	X	X
T cell analysis	10		X	X	X		X	X	X		X	X	X	X	X	X	X
Total Blood (mL) drawn		25	80	40	40	20	55	40	40	40	75	40	40	70	50	45	70

* - BV = Blood volume (mL) required for test. Total volume drawn over course of study is 770 mL.
** - CBC parameters to be assessed for safety: WBC, Hemoglobin, Platelet count,
*** - Female subjects only

6 ASSESSMENT OF OUTCOME / IMMUNE RESPONSE

6.1 OUTCOME PARAMETERS

The following parameters will be considered evidence of the efficacy of vaccination in inducing malaria-specific immune responses.

6.1.1 Serum antibody response to vaccination

6.1.2 Cellular immune response to vaccination

6.2 METHODS AND TIMING OF OUTCOME ASSESSMENTS

The following approach will be used to assess these parameters:

6.2.1 Antibody assay (ELISA):

Antibody levels to the AMA-1 antigen will be measured in serum by ELISA. Briefly, microwell plates (Dynex Technologies) are coated overnight at 4°C with 100 µl/well of antigen solution (1 µg/ml). Plates are washed with TRIS-buffered saline(TBS) containing 0.1% Tween-20 (0.1% T-TBS) and blocked with TBS containing 5% skim milk powder for two hours at room temperature. After washing with 0.1% T-TBS, serum samples in TBS containing 5% skim milk are added in triplicate and incubated with AMA-1-coated plates for 2 hours at room temperature. After incubation, unbound antibodies are removed by washing the plates with 0.1% T-TBS, and 100 µl of alkaline phosphatase-conjugated goat anti-human IgG solution (Kirkegaard & Perry Labs, Gaithersburg, MD, 1:1000 dilution in 0.5% T-TBS containing 1% BSA,) is added to each well and incubated for 2 hours at room temperature. Plates are then washed with 0.1% T-TBS, followed by adding 100 µL of substrate solution (Sigma 104 substrate, St. Louis, MO) to each well; the plates are then covered with aluminum foil and incubated for 20 minutes at room temperature for color development. The plates are read immediately at 405 nm with a microplate reader (Spectramax 340PC Molecular Devices). A serially diluted standard serum is run on each plate and will initially be obtained from a pool of human anti-AMA1 serum from malaria-endemic area of Mali. This serum is assigned a unit value as the reciprocal of the dilution giving an O.D. =1 on an AMA1-coated plate. Using the standard curve, the absorbance of individual test sera is converted to antibody units (SOFTmax PRO ver. 3; Molecular Devices Co.).

6.2.2 In vitro Parasite Growth inhibition assay (GIA):

Late trophozoite and schizont stages of *P. falciparum* parasitized erythrocytes are collected by Percoll gradient and/or 5% sorbitol treatment. The synchronized parasites are diluted with human RBCs to give a final concentration of 0.3% parasitemia, 1% hematocrit in growth medium (RPMI 1640 containing 10% human O+ serum, 25mM HEPES, 0.4mM hypoxanthine, 30mM sodium bicarbonate and 25mg/Li of gentamicin). Preliminary tests may be performed with test serum which has been heat inactivated (at 56ºC for 20 minutes) and pre-adsorbed with uninfected human O+ RBCs to remove anti-human immunoglobins. However, definitive studies are performed with IgG purified from human serum. Serial dilutions of immune serum or IgG are mixed with parasitized erythrocytes in 96-well tissue culture plates. Controls include autologous pre-immune serum (20%), human AB+serum (+), and uninfected erythrocytes (-). *P. falciparum* cultures are grown in 5% O_2, 5% CO_2, and 90% N_2 at 37°C for 40 hrs. After mixing, 50 µl samples are transferred to C-bottom 96-well plates containing 250 µl of cold PBS. Plates are then centrifuged to pellet the cells and frozen for at least 3hr to lyse the cells. Relative parasitemia levels are determined by means of a colorimetric measurement of parasite lactate dehydrogenase (LDH) activity. Test samples are dissolved in LDH substrate buffer (100uL of 100mM Tris,pH 8.0, containing 50 mM sodium L-lactate, 0.25% TritonX-100, 0.075mM 3-acetylpyridine adenine dinucleotide(APAD), 1U/ml diaphorase, 20 µg Nitro Blue Tetrazolium (all reagents from Sigma Chemical, St. Louis, MO)) and color allowed to develop in the dark for 30 minutes at room temperature. Absorbance at 650 nm is then determined using a Spectra Max 340PC (Molecular Devices) plate reader. Percent growth inhibition is calculated by the formula: 100% - [(A650 immune sample - A650 normal RBC only) / (A650 Pre-immune control - ABS650 RBC only) X 100%]. All assays are run in triplicate.

6.2.3 Assessment of cellular immune responses

Approximately 30 mL of whole blood will be obtained from subjects prior to vaccination and at time points after vaccination for assessment of B- and T-cell responses to AMA-1. These samples will be assayed for:

6.2.3.1 B Cell phenotype by flow cytometry:

AMA-1 specific activated B cells will be detected by staining with CD19, CD20, CD27, CD38, and Alexafluor-conjugated AMA-1. Results will be compared to those using fluorescently labeled BSA. Precise cell counts will be performed by Coulter counting to allow extrapolation of the FACS percentages to lymphocyte populations in peripheral blood of subjects.

6.2.3.2 Malaria-specific T cell effector cytokines:

Malaria-specific T cell responses will be evaluated by three single-color cytokine (IL2, IL4, IFN-γ) ELISPOT assays of PBL. In addition, FluoriSPOT assays using three-fluorescent dyes simultaneously will be performed in parallel in order to compare this assay, which has greater potential for automation and a larger dynamic range, with the ELISPOT results. In these assays AMA1-3D7 and AMA1-FVO will be used as antigens. If the frequency of T cell effectors is too high, the spot rate in diluted samples will be assessed, capturing B cell and naturally-adherent APC on the ELISPOT filters with anti-CD19 mAb to avoid dilutional effects in antigen presentation.

Finally, production of multiple cytokines and chemokines by antigen-specific T cells will also be assessed by in vitro stimulation of PBL with AMA1-FVO and AMA1-3D7 proteins using a quantitative multiplex bead ELISA (Luminex) system.

6.3 USE, STORAGE, AND TRACKING OF SPECIMENS AND DATA

Samples and data collected under this protocol will be used to study malaria and related diseases, and possible adverse reactions to vaccination. No genetic testing will be performed. Access to research samples will be limited using either a locked room or a locked freezer. Samples and data will be stored using codes assigned by the investigators or their designees. Data will be kept in password-protected computers. Only investigators or their designees will have access to the samples and data.

Samples will be stored at the MVDB in Rockville, MD or at MVDB's designated repository, Thermo Scientific, Rockville, MD. Samples will be tracked using a software database, e.g. Freezerworks. Any loss or unanticipated destruction of samples (for example, due to freezer malfunction) or data (for example, misplacing a printout of data with identifiers,) will be reported to the IRBs. Such a loss will be reported to the NIAID IRB as a protocol violation under the following classification: The violation compromises the scientific integrity of the data collected for the study.

6.4 RETENTION OF SPECIMENS FOR FUTURE USE

All specimens collected as part of this trial may, with the subject's permission, be stored for future research. Whether or not a volunteer agrees to storage of specimens will not affect his/her ability to participate in this trial. These samples may be used to learn more about malaria infection and other diseases. These samples will not be sold or used to make

commercial products. Samples will only be stored with the volunteer's permission. The volunteer may withdraw permission for future use of specimens at any time. If a volunteer withdraws his or her permission for future use of specimens, those specimens will be destroyed. All samples stored will be labeled with the volunteer's study identification (ID) number, which cannot identify the study subject but is linkable to other research databases (e.g., from questionnaires, clinical assessments, logbooks, etc.) generated by the main study. The database will contain only the study volunteer's ID number. A master log linking the study volunteer ID number to the name of the volunteer will be maintained in a password protected database system with access limited to authorized research team members.

At the completion of the protocol (termination), samples and data will either be destroyed, or after IRB approval, transferred to another existing protocol or a repository. In the future, other investigators (both at NIH and outside) may wish to study these samples and/or data. In that case, IRB approval must be sought prior to any sharing of samples. Any clinical information shared about the sample with or without patient identifiers would similarly require prior IRB approval. The research use of stored, unlinked or unidentified samples (for example, as a standard for immunological analyses), may be exempt from the need for prospective IRB review and approval. Exemption requests will be submitted in writing to the NIH Office of Human Subjects Research, which is authorized to determine whether a research activity is exempt.

7 ASSESSMENT OF THE SAFETY OF VACCINATION

Safety will be assessed by frequency and incidence of AEs and SAEs in each treatment group.

7.1 SAFETY PARAMETERS

Safety parameters will include diary cards which record symptoms from days 0-6 following each vaccine, vital signs at each study visit, recording of daily oral temperature for 6 days following vaccination, laboratory studies, and unsolicited adverse events.

7.2 METHODS AND TIMING OF SAFETY ASSESSMENTS

Subjects will be asked to keep daily symptom diaries recording oral temperature once daily, as well as pain, tenderness, redness, and induration at the injection site for six days

following each immunization. The size of any injection site reaction will be measured utilizing a standardized clear plastic measurement device and recorded in the volunteer symptom diary. See Appendix 17.4.

Using standard techniques, the clinical laboratory, will perform the following tests:

1. Complete blood count plus white blood cell differential (parameters to be assessed for safety: WBC, Platelet count, Hemoglobin)
2. Serum creatinine
3. Aspartate aminotransferase (AST)
4. Urinalysis (in the event of an abnormal urine dipstick test)
5. Rheumatoid factor - laboratory cut-off for negative results = “negative”
6. Anti-dsDNA (ELISA) – laboratory cut-off for negative result < 1:4
7. ANA – laboratory cut-off for negative result $\leq$ 1:80
8. C3
9. C4
10. CH50

Urine ß-hCG testing will be performed at the clinical trial site, using an FDA-approved urine pregnancy test kit. Urine dipstick testing will be performed at the clinical trial site using an FDA-approved product.

7.3 ADVERSE EVENTS, REACTOGENICITY, SERIOUS ADVERSE EVENTS

The Investigator is responsible for reporting all AEs that are observed or reported during the study, regardless of their relationship to study product. Initial vaccine reactions will be assessed at 30 minutes after vaccination following each vaccine dose. Subjects will be asked to record both solicited vaccine reactions (from 0 [not present] to 3+ [severe]) and any unsolicited AEs on a diary card for 6 days following both vaccinations.

Solicited systemic AEs will include the following: Feverishness/chills, malaise body aches (exclusive of the injection site), rash, loss of appetite, and headache.

Solicited injection site AE will include the following: Pain or tenderness, redness, and swelling.

7.3.1 Definition of Adverse Event

Adverse Event: ICH E6 Good Clinical Practice Guidelines defines an Adverse Event (AE) as any untoward medical occurrence in a patient or clinical investigation subject administered a pharmaceutical product regardless of its causal relationship to the study treatment. An AE can therefore be any unfavorable and unintended sign (including an abnormal laboratory finding), symptom, or disease temporally associated with the use of medicinal (investigational) product. The occurrence of an adverse event may come to the attention of study personnel during study visits and interviews or by a vaccine recipient presenting for medical care.

All adverse events must be graded for intensity and relationship to study product

Changes in the severity of an AE should be documented to allow an assessment of the duration of the event at each level of intensity to be performed. Adverse events characterized as intermittent require documentation of onset and duration of each episode.

Relationship to study products/vaccines: The investigator's assessment of an AE's relationship to study drug/vaccine is part of the documentation process, but it is not a factor in determining what is or is not reported in the study. If there is any doubt as to whether a clinical observation is an AE, the event should be reported. All adverse events must have their possible relationship to study vaccine assessed using the following terms: associated or not associated. In a clinical trial the study product must always be suspect. To help assess, the following guidelines are used.

- Associated – There is a known temporal relationship with study product administration and definite no other compelling etiology. Associated adverse events include those AEs judged to be definitely, probably, possibly, or unlikely related to vaccination.
- Not Associated -the AE is temporally independent of study product administration; and a compelling alternative etiology exists

Relationship of AEs to study product will be coded on a dual scale so that assignment of associated/not associated or definitely/probably/possibly/unlikely/not will both be captured and can be reported either way.

7.3.2 Reactogenicity Grading Scale

The grading scales for solicited clinical reactions, and for laboratory abnormalities, are given in appendix 17.2 and 17.3. Unsolicited clinical reactions will be graded according to appendix 17.5.

7.3.3 Definition of a Serious Adverse Event

A Serious Adverse Event is defined as an AE meeting one of the following conditions:

- Death during the period of protocol defined surveillance
- Life Threatening (defined as a subject at immediate risk of death at the time of the event)
- Requires inpatient hospitalization or prolongation of existing hospitalization during the period of protocol defined surveillance
- Results in congenital anomaly or birth defect
- Results in a persistent or significant disability/incapacity.
- Any other important medical event that may not result in death, be life threatening, or require hospitalization, may be considered a serious adverse experience when, based upon appropriate medical judgment, the event may jeopardize the subject and may require medical or surgical intervention to prevent one of the outcomes listed above. Examples of such medical events include allergic bronchospasm requiring intensive treatment in an emergency room or at home, blood dyscrasias or convulsions that do not result in inpatient hospitalization, or the development of drug dependency or drug abuse.

7.4 REPORTING PROCEDURES

Adverse Events including local and systemic reactions not meeting the criteria for "serious adverse events" will be captured on the appropriate case report form. Information to be collected includes event description, date of onset, investigator assessment of severity, relationship to study product, date of resolution of the event, seriousness, and outcome. All adverse events occurring while on study will be documented appropriately regardless of relationship. All AEs will be followed to adequate resolution.

Any medical condition that is present at the time that the patient is screened will be considered as baseline and not reported as an AE. If the severity of any preexisting medical condition increases during the study period, then it will be recorded as an AE.

7.4.1 Serious Adverse Event Detection and Reporting

All serious adverse events will be:

- recorded on the appropriate serious adverse event report form
- followed through resolution by a study physician
- reviewed by a study physician

Any AE considered serious by the Principal Investigator or Subinvestigator or which meets the aforementioned criteria must be submitted on an SAE form to PPD Development, NIAID's pharmacovigilance contractor, at the following address:

Medical Affairs/Pharmacovigilance

PPD Development

3151 17th St.

Wilmington, NC 28412

SAE Fax line: 888 488-9697

Questions about SAE reporting can be referred to the SAE Hotline (available 24 hours a day/7 days a week) at 800 201-8725

The study clinician will complete a Serious Adverse Event Form within the following timelines:

- All deaths, and all life-threatening events regardless of relationship, will be recorded on the Serious Event Form and sent by fax within 24 hours of site awareness of the death or life-threatening event.
- All other serious adverse events, regardless of relationship, will be reported via fax by the site within 72 hours of becoming aware of the event.

Other supporting documentation of the event may be requested by the pharmacovigilance contractor and should be provided as soon as possible.

All SAEs will be followed until satisfactory resolution or until the Principal Investigator or Subinvestigator deems the event to be chronic or the patient to be stable.

REGULATORY REPORTING FOR STUDIES CONDUCTED UNDER DMID SPONSORED IND

Following notification from the investigator, DMID, the IND sponsor, will report events that are both serious and unexpected that are related to study product(s) to the FDA within the required timelines as specified in 21 CFR 312.32: fatal and life threatening events within 7 calendar days (by phone or fax). All written reports will be sent within 15 calendar days. All serious events designed as "not related" to study product(s), will be reported to the FDA at least annually in a summary format.

REPORTING OF ADVERSE EVENTS TO THE NIAID IRB

Any AE considered serious by the Principal Investigator or Subinvestigator or which meets the aforementioned criteria will also be reported to the University of Rochester IRB and the NIAID IRB at 301-480-6606. A summary of data will be provided. Additionally, all Grade 3

and 4 adverse events that are determined to be associated with vaccination will be reported to the NIAID IRB within 15 days of the NIH investigator becoming aware of such events.

7.4.2 Reporting of Pregnancy

Pregnancies occurring in study participants will be reported by fax to the medical affairs/pharmacovigilance contractor at the address above. No further vaccinations will be administered to pregnant volunteers, but all study mandated blood samples will be obtained and the subject will continue in follow-up for safety events. Pregnancies will be followed for pregnancy outcome.

7.5 PROCEDURES TO BE FOLLOWED IN THE EVENT OF ABNORMAL LABORATORY TEST VALUES OR ABNORMAL CLINICAL FINDINGS

Laboratory Test Values: All laboratory test abnormalities will be reported to PPD and followed to resolution. The clinical significance of laboratory abnormalities will be assessed with appropriate referral for further workup as needed.

7.5.1 Procedures for subjects developing moderate or greater anemia

Subjects who develop decreased Hgb levels or changes in Hgb levels corresponding to toxicities graded as moderately severe or greater according to section 17.3 will have the following procedures:

- A workup, consisting of reticulocyte count, total iron binding capacity (TIBC), ferritin, direct Coombs test, haptoglobin level, total and direct bilirubin, and lactic acid dehydrogenase.
- Subjects found to be iron deficient will forgo further blood drawing for T cell (20 mL), B cell (10 mL) and GIA (20 mL) assays. Immunizations will continue and samples for laboratory safety tests and anti-AMA1 antibody will be obtained per schedule
- Subjects found to have autoimmune hemolytic anemia will not receive further doses of vaccine.

7.6 TYPE AND DURATION OF THE FOLLOW-UP OF SUBJECTS AFTER ADVERSE EVENTS

Adverse events will be followed until resolved or considered stable.

7.7 HALTING RULES

A Safety Monitoring Committee (SMC) will be asked to review cumulative safety data up to and including day 35 post-vaccination prior to dose escalation of the next cohort. If a dose of

vaccine is considered unacceptably reactogenic, dose escalation and/or additional vaccinations will be suspended until reviewed with the Safety Monitoring Committee and study sponsor. This decision will also be reported to the NIAID IRB. The SMC will adhere to DMID guidelines. If none of the above conditions occur, dose escalation will proceed.

The following criteria will be used to define unacceptable reactogenicity of the AMA1-C1/Alhydrogel® + CPG 7909 malaria vaccine:

1. One or more volunteers experience a Serious Adverse Event (SAE) that is determined to be associated (unlikely, possibly, probably, or definitely related) to the vaccination, OR

2. One or more volunteers experience anaphylaxis that is associated (unlikely, possibly, probably or definitely related) to the vaccine, OR

3. Any severe clinical illness occurs that is not explained by a diagnosis that is unrelated to vaccination, OR

4. One or more volunteers in a single-dose cohort experience an objective physical finding or laboratory abnormality of Grade 3 or higher, OR

5. Two or more volunteers in a single dose cohort experience a Grade 2 or higher laboratory abnormality or Grade 3 clinical AE.

The NIAID IRB will be notified of the decision to halt or to continue vaccinations within one working day of the NIH investigator becoming aware of such events.

8 CLINICAL MONITORING STRUCTURE

8.1 SITE MONITORING PLAN

Site Monitoring will be conducted to ensure that human subject protection, study procedures, laboratory procedures, study intervention administration, and data collection processes are of high quality and meet sponsor, GCP/ICH, and regulatory guidelines, and that the study is conducted in accordance with the protocol and sponsor SOPs. DMID, the sponsoring agency, or its designee will conduct site monitoring visits as detailed in the monitoring plan or in the Manual of Procedures.

Site visits will be made at standard intervals as defined by DMID and may be made more frequently as directed by DMID. Monitoring visits will include, but are not limited to, review of regulatory files, accountability records, case report forms, informed consent forms, medical and laboratory reports, and protocol compliance. Study monitors will meet with investigators to discuss any problems and actions to be taken and document visit findings and discussions.

9 BIOSTATISTICAL DESIGN AND ANALYSIS

9.1 STATISTICAL METHODS

This study, like other Phase 1 studies, is basically exploratory rather than confirmatory; its purpose is to estimate event rates and patterns of immune responses rather than to test formal statistical hypotheses. Estimates will be presented with their 95% confidence intervals. Descriptive approaches will be used to meet the protocol objectives as stated in this protocol, as well as formal statistical tests as outlined below. Results will be presented in tabular format, as well as graphically where appropriate.

9.2 ENDPOINTS

9.2.1 Safety

The primary safety endpoint is the frequency of vaccine-associated AEs, as classified by severity through active and passive surveillance. Separate assessments of systemic and local reactions will be performed.

9.2.2 Immunogenicity

The primary immunogenicity endpoint is antibody concentration as measured by ELISA at day 70. Anti-AMA1 antibody will be measured by ELISA on Days 0, 14, 28, 42, 56, 70, 84, 140, and 236 as listed in the schedule of visits.

9.3 ANALYSIS PLAN

Primary Objective 1: To assess the safety, reactogenicity and immunogenicity of the AMA1-C1/Alhydrogel® + CPG 7909 vaccine at each dose

a. Summarize the frequency of immediate, systemic, and local AEs.

b. Line listing of individual clinical and laboratory AEs as classified by immediate (within the first 30 minutes), systemic, and local will be displayed in tabular format and stratified by dose cohort.

c. AEs will be summarized by severity and relationship to vaccine by individuals and dose cohort.

Secondary Objective 1: To demonstrate that the addition of CPG 7909 improves the immune responses to AMA1-FVO and AMA1-3D7, as compared to AMA1-C1/Alhydrogel® at day 70.

a. Mann-Whitney tests will be used to compare the antibody concentrations between groups. Specifically, the subjects receiving 80 µg AMA1-C1/Alhydrogel® + CPG 7909 will be compared to those receiving only 80 µg AMA1-C1/Alhydrogel®.

Secondary Objective 2: To determine whether increasing dose increases the serum antibody levels to AMA1-FVO and AMA1-3D7 at day 70 in the AMA1-C1/Alhydrogel® + CPG 7909 groups.

a. A Mann-Whitney test will be performed on the two dose levels.

Secondary Objective 3: To assess and compare the duration of antibody responses to AMA1-FVO and AMA1-3D7.

a. Describe immunogenicity responses by dose, over time from ELISA data.

b. Individual responses will be described over time and stratified by dose cohort.

c. Analysis will be performed using longitudinal models.

Secondary Objective 4: To perform exploratory studies of B and T cell populations before and after vaccination

Other analyses:

1. To measure the inhibition of parasite growth as measured by the in vitro GIA to both FVO and 3D7 parasite clones.

a. Growth inhibition, expressed as a percent of inhibition comparing test sera to pre-immune sera, will be displayed graphically.

2. To determine the relationship between anti-AMA1 antibody concentration and degree of in vitro growth inhibition of *P. falciparum* in a GIA.

a. Rank correlation between ELISA values and growth inhibition will be assessed by the Spearman Rank correlation test.

b. Growth inhibition values will be plotted graphically as a function of ELISA units of antibody of each serum and stratified by dose.

3. To determine the relative specificity of the antibodies to a range of AMA1 serotypes, in addition to FVO and 3D7, as judged by ELISA, and growth inhibition on a select panel of parasites with typed AMA1.

In addition, models that account for correlation of responses within volunteer (such as marginal models or mixed models) will be used to study the immune responses. The approaches to missing data and the particular methods for modeling will be described in the analysis. Listings will show the observed data and, if applicable, imputed values and the approaches taken for imputation.

Should the need arise for terminating the study early, the investigative team will discuss with the SMC the reason for termination and determine which study questions can be addressed in an unbiased manner with the available data. The available data will be analyzed and interpreted in light of early termination.

9.4 SAMPLE SIZE

A group size of 15 subjects per dose gives a probability of about 0.8 for detecting one or more serious or severe AE that occur with a frequency of 0.1 per volunteer.

Based on an analysis of the human antibody responses to a number of malaria antigens that have been tested in clinical trials [7, 8], the observed coefficient of variation in the range of antibody concentrations has been found to be remarkably constant at approximately 1.2 - 1.4. Assuming the larger coefficient of variation and assuming that one group has mean antibody concentrations that are at least 2.2 times the mean antibody concentrations of the other group, then the two-sided Mann-Whitney test assuming a significance of 0.05 performed on the sample size of 30 per group (see Secondary Objective 1 above) will have a power of greater than 0.80.

9.5 MISSING DATA

Missing data will not be replaced.

9.6 DEVIATIONS FROM THE STATISTICAL PLAN

Deviations from the statistical plan will be reported in the final study report.

9.7 SELECTION OF SUBJECTS FOR ANALYSIS

All enrolled subjects will be included in the data analysis.

10 DIRECT ACCESS TO SOURCE DATA/DOCUMENTS

The trial site will maintain appropriate medical and research records for this trial, in compliance with ICH E6 GCP, Section 4.9 and regulatory and institutional requirements for the protection of confidentiality of subjects. The site will permit authorized representatives of the DMID, its designees, and appropriate regulatory agencies to examine (and when required by applicable law, to copy) clinical records for the purposes of quality assurance reviews, audits and evaluation of the study safety and progress. These representatives will be permitted access to all source data.

11 QUALITY CONTROL AND QUALITY ASSURANCE

Following written standard operating procedures, the monitors will verify that the clinical trial is conducted and data are generated, documented (recorded), and reported in compliance with the protocol, GCP, and the applicable regulatory requirements. Reports will be submitted to DMID on monitoring activities.

The investigational sites will provide direct access to all trial-related sites, source data/documents, and reports for the purpose of monitoring and auditing by the sponsor, and inspection by local and regulatory authorities.

The Data Coordinating Center (DCC) will implement quality control procedures beginning with the data entry system and generate data quality control checks that will be run on the database. Any missing data or data anomalies will be communicated to the site(s) for clarification and resolution.

12 ETHICS/PROTECTION OF HUMAN SUBJECTS

12.1 Declaration of Helsinki

The investigator will ensure that this study is conducted in full conformity with the current revision of the Declaration of Helsinki, or with the International Conference for Harmonisation Good Clinical Practice (ICH-GCP) regulations and guidelines, whichever affords the greater protection to the subject.

12.2 Institutional Review Board

Prior to enrollment of subjects into this trial, the approved protocol and the informed consent form will be reviewed and approved by the appropriate Institutional Review Board (IRB).

The responsible official for the IRB will sign the IRB letter of approval of the protocol prior to the start of this trial and a copy will be provided to DMID. Notification of the IRB's composition, or the IRB’s MPA or FWA number, will be provided to DMID.

Should amendments to the protocol be required, the amendments will be written by the sponsor and provided to the investigator for submission to the IRB.

12.3 Informed Consent Process

The investigator will choose subjects in accordance with the eligibility criteria detailed in section 4. The investigator will not exercise selectivity so that bias is prevented. All subjects must sign an informed consent form that complies with the requirements of both 21 CFR Part 50 and HIPAA before entering the trial. Or, a consent form that complies with the requirements of 21 CFR Part 50 and a separate HIPAA compliant authorization form for the use and disclosure of the subject's protected health information (PHI) may be used per institutional standard operating procedures.

Prior to the trial, subjects will receive a comprehensive explanation of the proposed treatment including the nature and risks of the trial, alternate therapies, any known adverse events, the investigational status of the components, and the other elements that are part of obtaining proper informed consent. Subjects will also receive a detailed explanation of the proposed use and disclosure of their protected health information, including specifically their serum samples. Subjects will be allowed sufficient time to consider participation in the trial, after having the nature and risks of the trial explained to them. The consent form must not include any exculpatory statements. This consent form and any separate HIPAA authorization form, if applicable.

The Principal Investigator and study collaborators, in cooperation with the study sponsor (DMID), will provide to subjects any new information that significantly affects the risk or benefit to continued participation in this study. This new information will be communicated by the investigator to subjects who consent to participate in the trial in accordance with IRB requirements. The informed consent will be updated and subjects will be reconsented, if necessary.

Site staff may employ recruitment efforts prior to the subject consenting; however, before any protocol-specific procedures are performed to determine protocol eligibility an informed consent form must be signed. Subjects will be given a copy of all consent forms that they sign.

By signing the informed consent form, the subject agrees to complete all evaluations required by the trial, unless the subject withdraws voluntarily or is terminated from the trial for any reason.

12.4 Exclusion of Women, Minorities, and Children (Special Populations)

This study will be inclusive of all healthy adults who meet the inclusion/exclusion criteria, regardless of religion, sex, or ethnic background. Since this study is greater than minimal risk and without direct benefit to the subject, children will not be enrolled. Only individuals who are between the ages of 18 and 45 years of age will be included at this time. Should the outcome

of this study be deemed acceptable, additional trials of the vaccine in both younger (infants, children, and minors) and older (46 years or older) populations will be initiated.

12.5 SUBJECT CONFIDENTIALITY

Volunteers will have code numbers and will not be identified by name. Subject confidentiality is strictly held in trust by the participating investigators, their staff, the sponsor(s), and their agents. This confidentiality extends to genetic and biological sample tests, in addition to the clinical information relating to participating subjects.

The study protocol, documentation, data and all other information generated will be held in strict confidence. No information concerning the study or the data will be released to any unauthorized third party without prior written approval of the sponsor.

The study monitor or other authorized representatives of the sponsor may inspect all documents and records required to be maintained by the Investigator. This includes, but is not limited to, medical records (office, clinic or hospital) and pharmacy records for the subjects in this study. Clinical study sites will permit access to such records.

13 A HANDLING AND RECORDKEEPING

The investigator is responsible to ensure the accuracy, completeness, legibility and timeliness of the data reported.

13.1 SOURCE DOCUMENTS AND ELECTRONIC CASE REPORT FORMS:

Copies of the electronic case report forms will be provided for use as source documents and maintained for recording data for each subject enrolled in the study. All source documents should be completed in a neat, legible manner to ensure accurate interpretation of data. Black ink is required to ensure clarity of reproduced copies. When making a change or correction, cross out the original entry with a single line and initial and date the change. DO NOT ERASE, OVERWRITE OR USE CORRECTION FLUID OR TAPE ON THE ORIGINAL.

Date reported in the eCRF derived from source documents should be consistent with the source documents or the discrepancies should be explained.

The sponsor will provide guidance to Investigators on making corrections to the source documents and eCRFs. .

13.2 DATA MANAGEMENT RESPONSIBILITIES

All source documents and laboratory reports must be reviewed by the clinical team and data entry staff, who will ensure that they are accurate and complete. Adverse Events must be graded, assessed for severity and causality and reviewed by the site Principal Investigator or designee. Data collection is the responsibility of the clinical trial staff at the site under the supervision of the site Principal Investigator. During the study, the Investigator must maintain complete and accurate documentation for the study.

The EMMES Corporation will serve as the Statistical and Data Coordinating Center for this study, and will be responsible for data management, quality review, analysis and reporting of the study data.

13.3 Data Capture Methods

Clinical data (including AEs, concomitant medications, and reactogenicity data) will be entered into a 21CFR11-compliant Internet Data Entry System provided by The EMMES Corporation. The data system includes password protection and internal quality checks, such as automatic range checks to identify data that appear inconsistent, incomplete, or inaccurate. Clinical data will be entered directly from the source documents.

13.4 Types of Data

Data for this study will include safety, laboratory (immunologic and clinical) and outcome measures (e.g., reactogenicity, immunogenicity, clinical).

13.5 Timing/Reports

Safety data for Stage 1 will be reviewed by the SMC after the collection of the Day 7 and Day 35 study visits (7 days after the first and second vaccinations). The SMC will make a recommendation at that time as to the advisability of proceeding with the vaccination of the Stage 2 subjects. Similarly, safety data for Stage 2 will be reviewed after collection of day 7 and 35 study visits and reviewed prior to initiation of stage 3.

13.6 Study Records Retention

Records and documents pertaining to the conduct of this study, including CRFs, source documents, consent forms, laboratory test results and medication inventory records, must be retained by the investigator for at least 15 years. No study records shall be destroyed without prior authorization from NIAID.

13.7 Protocol Deviations

The investigator will not deviate from this protocol for any reason without prior verbal or written approval from the sponsor, except in cases of medical emergencies. The investigator may deviate from the protocol without prior approval only when the change is necessary to eliminate an apparent and immediate hazard to the subject. In that event, the investigator will notify the sponsor immediately by phone, notify the IRB and confirm notification to the sponsor in writing within 5 working days after the change is implemented.

14 FINANCING AND INSURANCE

This study is supported by a contract between NIH and the University of Rochester

15 PUBLICATION POLICY

It is the intention that the results of this study will be published in a peer-reviewed journal readily accessible to the medical community. The specific publication policy is described in the Manual of Procedures. All publications will be submitted to DMID prior to journal submission.

16 LITERATURE CITED

1. World Health Organization: Tropical Disease Research. World Health Organization, 2002. (Accessed August 20, 2004, at http://www.who.int/tdr/diseases/malaria/diseaseinfo.htm.)
2. Narum DL, Thomas AW. Differential localization of full-length and processed forms of PF83/AMA1 , an apical membrane antigen of *Plasmodium falciparum* merozoites. *Molecular and Biochemical Parasitology* 1994;67:59-64.
3. Crewther PE, Culvenor JG, Silva A, Cooper JA, Anders RF. Plasmodium falciparum: Two antigens of similar size are located in different compartments of the rhoptry. *Experimental Parasitology* 1990;70:193-206.
4. Waters AP. A merozoite receptor protein from *Pasmodium knowlesi* is highly conserved and distruibuted throughout Plasmodium. *J Biol Chem* 1990;265:17974-9.
5. Peterson MG. Integral membrane protein located in the apical complex of Plasmodium falciparum. *Molecular Cell Biology* 1989;9:3151-4.
6. Recombivax HB product insert. 1999. (Accessed February 14, 2002, at http://www.whale.to/m/recombivax_hb.html.)
7. Saul A, Lawrence G, Smillie A. Human Phase I vaccine trials of 3 recombinant asexual stage malaria antigens with Montanide ISA 720 adjuvant. *Vaccine* 1999;17:3145-59.
8. Stoute JA, Slaoui M, Heppner G, Momin P, Kester KE, Desmons P, Wellde BT, Garcon N, Krzych U, Marchand M, Ballou WR, Cohen JD. A preliminary evaluation of a recombinant circumsporozoite protein vaccine against *Plasmodium falciparum* malaria. *N Engl J Med* 1997;336(1):86-91.

17 APPENDICES

17.1 SUMMARY OF PRECLINICAL ANIMAL STUDIES

Study #	Purpose	Species	Dose & Regimen	Formulations Tested	Summary
AP-5063	Comparative Immunogenicity of AMA1-C1 formulated with Alum alone and Alum + CPG 7909 in mice	BALB/c mice (n=80)	0.003, 0.01, 0.03, 0.1 µg Days 0 & 28 IM	Alhydrogel & Alhydrogel + 20 µg CPG 7909	Animals that received either the 0.03 or 0.1 µg of AMA1-C1/Alhydrogel + CPG 7909 made significantly greater immune responses than those that received the formulation without CPG 7909. No clinically significant adverse effects reported.
AP-5064	Comparative Immunogenicity of AMA1-C1 formulated with Alhydrogel and Alhydrogel + CPG 7909s in rats	Charles River - Sprague Dawley Rats (n=30)	0.3, 1, 3 µg on Days 0 & 28 IM	Alhydrogel & Alhydrogel + 200 µg CPG 7909	Immune responses were significantly greater at each antigen dose in animals that received the CPG 7909 formulation. No clinically significant adverse effects reported.
AP-5065	Immunogenicity of AMA1-C1/Alhydrogel +CPG 7909 and AMA1-C1/Alhydrogel in Guinea Pigs	Guinea Pig (n=30)	0.3, 1, 10 µg on Days 0 & 28 IM	Alhydrogel & Alhydrogel + 200 µg CPG 7909	The immune response was significantly greater in animals that received the CPG 7909 formulation at all 3 doses tested. No clinically significant adverse effects reported.

17.2 TOXICITY TABLES FOR SOLICITED ADVERSE EVENT INTENSITY

Adverse event	Mile (Grade 1)	Moderate (Grade 2)	Severe (Grade 3)
Pain at injection site	Easily tolerated	Interferes with daily activity	Prevents daily activity
Erythema at injection site	>0 to ≤ 20 mm	>20 to ≤ 50 mm	> 50 mm
Swelling at injection site	>0 to ≤ 20 mm	>20 to ≤ 50 mm	> 50 mm
Fever (oral)	> 37.6° to 38°C	> 38°C to 39°C	> 39°C
Headache	Easily tolerated	Interferes with daily activity	Prevents daily activity
Nausea	Easily tolerated	Interferes with daily activity	Prevents daily activity
Myalgia	Easily tolerated	Interferes with daily activity	Prevents daily activity
Arthralgia	Easily tolerated	Interferes with daily activity	Prevents daily activity
Urticaria	Requires no mediqations	Requires oral or topical treatment, or IV therapy or steroids for less than 24 hours	Requires IV therapy, or steroids for more than 24 hours

17.3 Toxicity Grading Scale for Laboratory Adverse Events

Laboratory	Mild (Grade 1)	Moderate (Grade 2)	Severe (Grade 3)
Hgb (♀) – gm/dl	< 11.5 & ≥ 11.0	< 11.0 & ≥ 10.0	< 10.0
Hgb (♀) – change from baseline value in gm/dl	≥ 1.0 & < 1.5	≥ 1.5 & < 2.0	≥ 2.0
Hgb (♂) – gm/dl	< 12.5 & ≥ 12.0	< 12.0 & ≥ 11.0	< 11.0
Hgb (♂) – change from baseline value in gm/dl	≥ 1.5 & < 2.0	≥ 2.0 & < 2.5	≥ 2.5
WBC – cells/mm^3 (Increase in WBC)	≥ 11000 & < 15000	≥ 15000 & < 20000	≥ 20000
WBC – cells/mm^3 (Decrease in WBC)	< 3500 & ≥ 2500	< 2500 & ≥ 1500	< 1500
Platelets – cell/mm^3	< 135,000 & ≥ 125,000	< 125,000 & ≥ 100,000	< 100,000
AST/ALT (increase by factor)	> 1.0 & < 2.5 x ULN	≥ 2.5 & < 4 x ULN	≥ 4 x ULN
Serum creatinine – mg/dL	IN* - IN+0.2	> IN+0.2 - < 2.0	≥ 2.0

URINALYSIS				
	Grade 1	Grade 2	Grade 3	Grade 4
Proteinuria	2+ or 0.5 - 1 gm loss/day	3+ or 1 - 2 gm loss/day	4+ or 2 - 3.5 gm loss/day	nephrotic syndrome or > 3.5 gm loss/day
Hematuria	5-10 rbc/hpf	>10 rbc/hpf	gross, with or without clots, OR red blood cell casts	requires hospitalization

Appendix 17.4 – Subject Diary Card and Injection Site Measurement Tool

17.5 Common Toxicity Criteria for Grading Unsolicited Adverse Events
